# Responsive hydrogel delivery of DFO-programmed apoptotic bodies drives redox-immuno-angiogenic remodeling in diabetic wounds

**DOI:** 10.1186/s12951-026-04817-0

**Published:** 2026-07-22

**Authors:** Hao Guo, Jian Li, Chengcheng Gu, Ning Liu, Jie Sun, Danhui Wang, Siyuan Meng, Dongshu Geng, Yunfei Lian, Fangnan Lv, Meirong Huo

**Affiliations:** 1 Department of Pharmaceutics, China Pharmaceutical University, Nanjing, 211198 China; 2 Nanjing Sairicon Biomedical Technology Co., Ltd, Nanjing, 210039 China

**Keywords:** Exosomes, DFO-programmed apoptotic bodies, Transcriptomic reprogramming, Tissue regeneration, Redox-immuno-angiogenic synergy

## Abstract

Mesenchymal stem cell (MSC)-derived exosomes show clear potential in cell-free regenerative medicine. However, their clinical translation is limited by low production yields and inconsistent therapeutic efficacy. To address this, we actively programmed stem cell apoptosis to generate high-yield, functional extracellular vesicles. By using deferoxamine (DFO) to stabilize HIF-1α prior to UV-induced apoptosis, we produced DFO-programmed apoptotic bodies (DABs). These programmed apoptotic bodies demonstrated a significantly higher production yield than conventional exosomes. Multi-omics analyses revealed that DABs inherit an enriched, pro-regenerative miRNA repertoire that functionally outperforms both native apoptotic bodies and exosomes. Beyond promoting classical angiogenic and migratory signaling, DABs support tissue repair by altering the mitochondrial bioenergetics of recipient cells. To prevent rapid in vivo clearance and address the highly oxidative diabetic microenvironment, we encapsulated DABs within a glucose- and ROS-responsive hydrogel, developing the DABs@PHA-PVA^Gel^. In vivo transcriptomics demonstrated that DABs@PHA-PVA^Gel^ synchronized active ROS scavenging with the on-demand release of DABs. This localized delivery improved complete tissue reconstruction by simultaneously enhancing the Cxcr4/Nrp1/S1pr1 angiogenic axis, activating the Daglb/Cnr2 anti-inflammatory pathway, and engaging the Txnip/Foxo4/Nrf2 antioxidant cascade. Overall, this study establishes programmed apoptotic bodies as a scalable and effective alternative to standard exosome therapies for tissue regeneration.

**Graphical abstract:**

**Figure:**
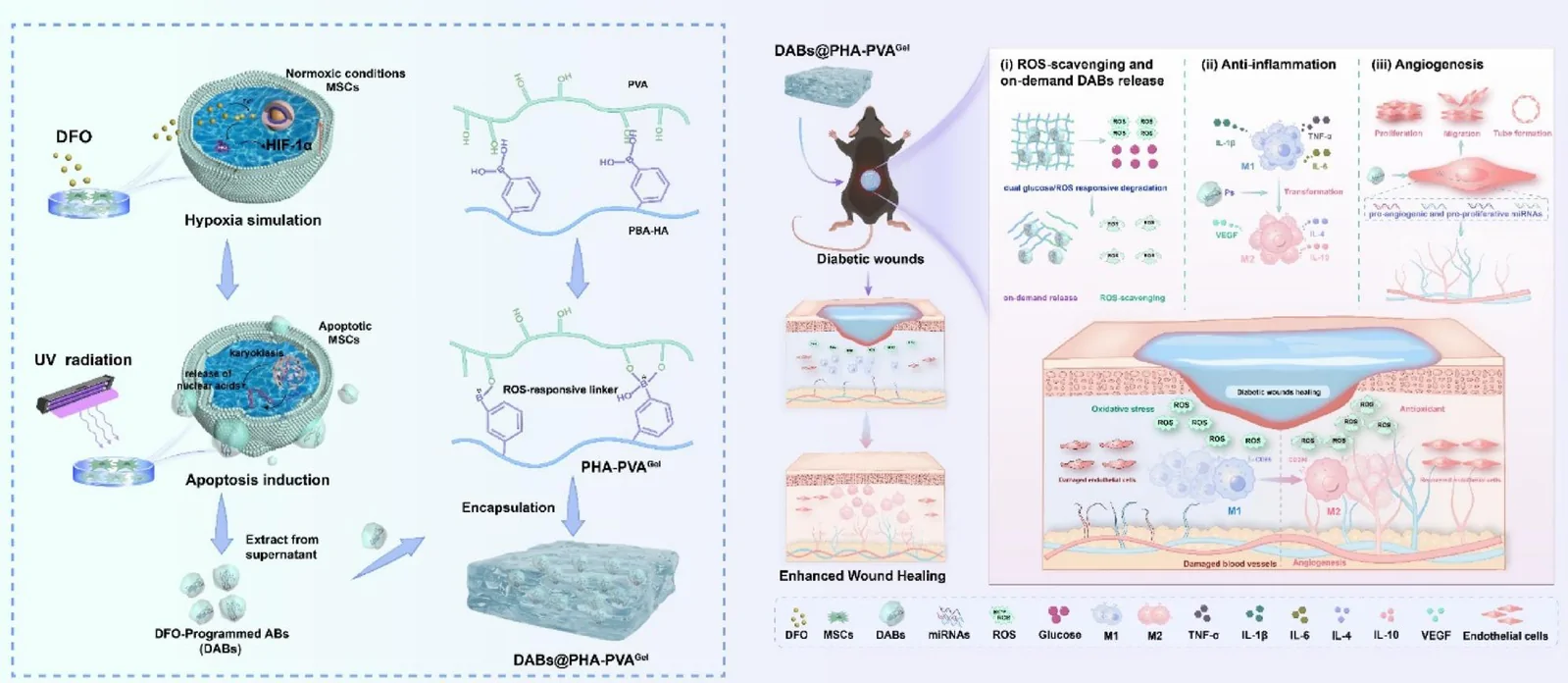

**Supplementary Information:**

The online version contains supplementary material available at https://doi.org/10.1186/s12951-026-04817-0.

## Introduction

Mesenchymal stem cell-derived extracellular vesicles (MSC-EVs), particularly exosomes, have attracted growing interest as cell-free therapeutics capable of delivering bioactive cargoes to injured tissues [1–3]. Despite these promising features, clinical translation of MSC-EVs remains limited. Exosome biogenesis is inherently low yield, and even when enough are obtained, their cargo composition is often inconsistent and insufficiently potent for use in diseased tissues [4, 5]. These persistent problems have pushed researchers to look beyond exosomes for better-suited EV sources.

Apoptotic bodies (ABs) were long dismissed as inert cellular debris from programmed cell death, a view now challenged by their proven biological and therapeutic activities [6, 7]. Apoptosis naturally generates these vesicles in massive quantities. Mechanistically, this high-yield vesicle generation differs from the constitutive secretion of conventional exosomes. Exosomes are mainly generated through the endosomal multivesicular body pathway and are continuously secreted by viable cells at relatively limited levels [8]. In contrast, apoptosis triggers a coordinated cellular disassembly program involving plasma membrane blebbing, apoptotic membrane protrusion formation, and fragmentation of dying cells into numerous membrane-bound apoptotic vesicles. This process can package substantial portions of the plasma membrane, cytoplasm, and cellular components into extracellular vesicles, thereby providing a more abundant vesicle source than steady-state exosome secretion [9]. They characteristically expose phosphatidylserine (PS) on their surface to act as an “eat-me” signal, which facilitates macrophage clearance and drives anti-inflammatory responses [10]. This combination of high yield and immune modulation makes them an attractive alternative to conventional exosomes [11]. Recent studies have further demonstrated the therapeutic relevance of stem cell-derived apoptotic bodies in diabetic wound healing. For example, adipose stem cell-derived apoptotic bodies were reported to accelerate diabetic wound repair by balancing macrophage polarization, while lysosomally escaped apoptotic bodies were shown to suppress inflammasome synthesis in macrophages [12, 13]. However, a critical limitation is that native ABs are insufficient at promoting new blood vessel formation—a prerequisite for repair in ischemic or poorly vascularized tissues [14, 15]. This gap in pro-angiogenic activity limits their utility in more demanding pathological settings, and to date, few studies have demonstrated a reliable strategy to overcome it.

We reasoned that this limitation might be overcome not by modifying the vesicles themselves, but by reprogramming the cells that produce them. Specifically, we pretreated MSCs with deferoxamine (DFO), an FDA-approved iron chelator with a well-established clinical safety record [16], prior to apoptosis induction. DFO stabilizes HIF-1α by blocking its iron-dependent prolyl hydroxylase-mediated degradation [17, 18], effectively placing the parent cells in a sustained hypoxia-mimicking state prior to undergoing apoptosis. Such HIF-1α-related programming can influence the transcriptional and post-transcriptional state of parental cells and has been associated with altered EV release and RNA cargo composition [19]. Therefore, we hypothesized that DFO pretreatment could reshape the regulatory cargo of the resulting apoptotic bodies by altering the biological state of parental MSCs before apoptosis induction. The apoptotic bodies generated from these programmed cells, which we term DFO-programmed apoptotic bodies (DABs), are consequently loaded with a markedly enriched profile of pro-regenerative miRNAs compared to their native counterparts. The approach shifts the therapeutic intervention upstream to the cell itself rather than engineering the vesicles post-isolation.

Importantly, DFO was used to program parental MSCs before apoptosis rather than to generate DFO-loaded apoptotic bodies. Compared with conventional MSC-derived exosomes, DABs offer two conceptual and mechanistic advantages. First, apoptosis is a robust vesiculation process that generates apoptotic vesicles in substantially higher quantities than constitutive exosome secretion, providing a more scalable source of therapeutic vesicles. Second, apoptotic bodies retain phosphatidylserine-rich membrane features, which may promote macrophage recognition, efferocytosis, and immunoregulatory responses. Nevertheless, native apoptotic bodies alone are not optimized for the strong pro-angiogenic and metabolic demands of diabetic wound repair. In this study, DFO preconditioning was therefore introduced as an upstream programming strategy to alter the biological state of parental MSCs before apoptosis induction. By stabilizing HIF-1α-related signaling, DFO reshaped the vesicular miRNA cargo of DABs toward angiogenesis, cell migration, VEGF-related signaling, cytoskeletal remodeling, and mitochondrial bioenergetic regulation. Thus, DABs are designed to integrate the scalability and immunomodulatory features of apoptotic bodies with the enhanced pro-regenerative cargo induced by DFO programming, distinguishing them from both conventional exosomes and native ABs.

To test DABs under genuinely demanding conditions, we turned to the diabetic wound model. These wounds are notoriously difficult to treat, owing to the combined burden of dysregulated inflammation, poor perfusion, and oxidative stress [20–22]. Given the central role of macrophage phenotype transitions in wound repair, immunomodulatory biomaterials and hydrogel systems capable of regulating macrophage polarization have emerged as promising strategies for improving chronic wound healing [23]. From a vesicle delivery standpoint, this environment is particularly hostile: elevated proteolytic activity and ROS can structurally compromise EVs membranes, while wound exudate rapidly flushes away unprotected cargo before it can act [24, 25]. To address this, we encapsulated DABs in a glucose- and ROS-responsive hydrogel (PHA-PVA^Gel^) that doubles as a local ROS scavenger, enabling sustained on-demand release directly at the wound site [26].

**Fig. 1:**
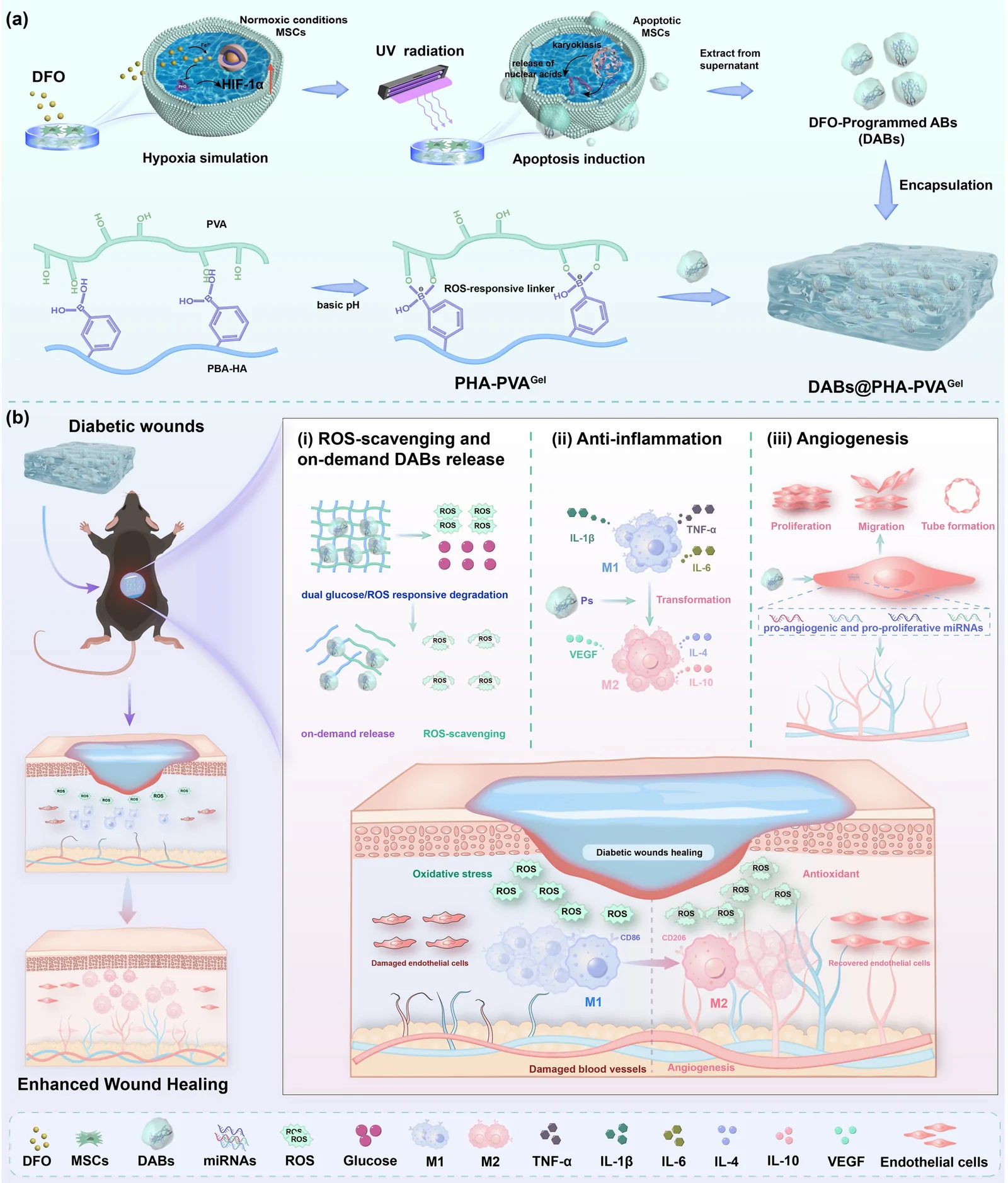
Schematic illustration of DABs@PHA-PVA^Gel^ for promoting diabetic wound healing. (**a**) Preparation of DFO-programmed apoptotic bodies (DABs) and their encapsulation in the glucose/ROS-responsive PHA-PVA^Gel^. (**b**) Proposed mechanism of DABs@PHA-PVA^Gel^ in diabetic wound healing, highlighting ROS scavenging with DABs on-demand release, macrophage polarization, and angiogenesis

In this study, we demonstrate that DABs with DFO-remodeled miRNA cargo display enhanced immunomodulatory and pro-angiogenic activity compared with native ABs and conventional exosomes in the tested in vitro and free-vesicle settings. Unexpectedly, this miRNA-enriched cargo profile was associated with pathways related to mitochondrial function in recipient cells, enhancing ATP output. By integrating these miRNA optimized DABs with a ROS-scavenging hydrogel, we successfully translated these cellular advantages into tissue regeneration within a highly hostile diabetic in vivo model. Together, these findings support programmed apoptotic bodies as a scalable and mechanistically distinct vesicle platform that may complement conventional exosome-based strategies for tissue repair (Fig. 1a and b).

## Materials and methods

### Materials

Deferoxamine mesylate (DFO) was obtained from Abcam plc. Hyaluronic acid (HA) was purchased from Bloomage Biotech Co., Ltd. Human albumin (HSA) was purchased from ExCell Biotechnology, Sexton Biotechnology, and Grifols, respectively. High-glucose DMEM and DMEM/F12 basal media were purchased from Jiangsu KeyGEN BioTECH. For cell viability and apoptosis analyses, the AO/PI double staining kit was acquired from APExBIO Technology, while the Coomassie brilliant blue and cell apoptosis kits were from YEASEN Biotechnology. A comprehensive panel of biochemical reagents and antibodies was purchased from Beyotime Biotechnology; this included the BCA protein assay kit, BeyoExo™ Exosome Identification Kit (CD63, CD9, TSG101, Hsp70, and Calnexin), DiI/DiO lipophilic dyes, as well as all primary and secondary antibodies for immunofluorescence and immunoblotting (anti-HIF-1α, anti-CD86, anti-CD206, anti-CD31, anti-CD105, anti-VEGF-A, anti-TGF-β, anti-α-SMA, HRP-conjugated β-Actin, Anti-Rabbit IgG (H + L), and AF 488/647). Mouse ELISA kits for inflammatory and angiogenic cytokines were obtained from MULTI SCIENCES. Specific antibodies for flow cytometry were sourced from BD Pharmingen.

### Cell culture

Human umbilical vein endothelial cells (HUVEC), human keratinocyte cells (HaCaT), and mouse macrophage cell line (RAW264.7) were purchased from the Stem Cell Bank, Chinese Academy of Sciences (Shanghai, China). They were cultured in DMEM medium containing 10% FBS in a 37°C, 5% CO_2_ incubator. Specifically, human umbilical cord mesenchymal stem cells (MSCs) were kindly provided by Jiangsu Province Cell Therapy Manufacturing Center (Nanjing, China) and previously identified, were cultured in DMEM/F12 containing 5% human platelet lysate (Sexton) in a 37°C, 5% CO_2_ incubator. The immunophenotype of MSCs was verified by flow cytometry using antibodies against CD45, HLA-DR, CD73, and CD105, with corresponding isotype controls.

### Animal

Male C57BL/6 mice (6 weeks of age, 18–22 g) were sourced from Jiangsu Huachuang Sino Pharmaceutical Technology Co., Ltd. (Taizhou, China). Animal housing and care were managed by the Animal Experimental Center of China Pharmaceutical University. All in vivo procedures received ethical approval from the Ethics Committee of China Pharmaceutical University (Nanjing, China) and were executed in full compliance with institutional guidelines for animal care.

### Optimization of DFO concentration for MSC programming

To determine the optimal DFO concentration for parental MSC programming, MSCs were treated with different concentrations of DFO (0, 150, 200, 250, 300, and 350 µg/mL) for 24 h. After treatment, cells were washed with PBS and collected for Western blot analysis to evaluate HIF-1α expression. To further determine whether DFO-induced HIF-1α stabilization remained detectable after apoptosis induction, MSCs were pretreated with the same concentration gradient of DFO for 24 h, washed with PBS, subjected to UV irradiation for 10 min, and then cultured in HPL-free medium for 24 h under the same apoptosis-induction conditions used for DABs generation. After 24 h of apoptosis induction, cell lysates were collected for Western blot analysis of HIF-1α expression. For Western blot analysis, cells were lysed using RIPA buffer containing protease inhibitors. The protein concentration was determined using a BCA protein assay kit. Equal amounts of protein were separated by SDS-PAGE and transferred onto PVDF membranes. After blocking, the membranes were incubated with primary antibodies against HIF-1α and the corresponding loading control proteins, followed by HRP-conjugated secondary antibodies. Protein bands were visualized using enhanced chemiluminescence, and relative HIF-1α expression was quantified using ImageJ. β-Actin or GAPDH was used as the loading control.

### Apoptosis induction of MSCs

Based on the DFO concentration optimization results, 250 µg/mL DFO was selected for subsequent parental MSC programming. MSCs at passages fewer than six were cultured in DMEM/F12 with 5% HPL containing DFO (250 µg/mL) or PBS for 24 h. The priming medium was then discarded, and the cells were rinsed three times with PBS before HPL-free medium was added. In this study, DFO was used as an upstream parental-cell programming cue rather than as a directly encapsulated vesicular cargo. Therefore, the resulting DABs were generated from DFO-preconditioned MSCs after the removal of DFO-containing medium. The confluent monolayers of DFO-MSCs and MSCs, maintained in uncovered cell culture dishes, were irradiated with ultraviolet light (UV) (30 W, 254 nm, Philip, China) at an intensity of 300 mJ/cm² for 10 min. After 24 h of apoptosis induction, apoptosis was evaluated by morphological observation and Annexin V-FITC/PI staining. In addition, apoptotic collapse in apoptotic DFO-MSCs (denoted as Apo-D-MSCs) and apoptotic MSCs (denoted as Apo-MSCs) was observed using inverted fluorescence microscopy. By contrast, confocal laser scanning microscopy (LSM800, Zeiss) was employed to monitor the generation of apoptotic vacuoles within DiI-stained MSCs.

### Isolation of apoptotic bodies

Apoptotic bodies were extracted from the culture supernatant of Apo-D-MSCs and Apo-MSCs using a widely used differential centrifugation protocol (denoted as DABs and ABs). Briefly, after 24 h UV irradiation induction, the HPL-free cell culture supernatant of Apo-D-MSCs and Apo-MSCs were collected and centrifuged at 400×g for 5 min to precipitate dead cells, and at 2000×g for 30 min to precipitate cell debris. Following the removal of dead cells and larger debris, the clarified supernatant was subjected to centrifugation at 40,000 × g for 40 min to sediment the apoptotic vesicles. To clear residual co-precipitating proteins, these crude pellets were washed by resuspension in ice-cold PBS and re-pelleted under identical centrifugation conditions. The final ABs and DABs preparations were then dispersed in 100 µL of PBS and preserved at − 80 °C for downstream applications.

### Characterization of DABs and ABs

Total protein concentrations of DABs and ABs were measured by a bicinchoninic acid (BCA) assay. Particle size distribution and zeta potential were measured by a Zetasizer Nano (Nano-ZS90, Malvern, UK). Morphology was checked using transmission electron microscopy (TEM). Total protein from MSCs, Apo-D-MSCs, Apo-MSCs, ABs, and DABs was extracted with RIPA buffer. This protein was mixed with loading buffer and heated at 95 °C for 5 min for SDS-PAGE. The protein bands were stained with Coomassie Brilliant Blue and photographed. Surface phosphatidylserine (PS) on DABs and ABs was measured by Annexin V-FITC labeling and flow cytometry. For comparative Western blot analysis of exosome-associated markers, equal amounts of total vesicular protein from Exo, ABs, and DABs were loaded in each lane, and Hsp70, CD63, TSG101, CD9, and Calnexin were detected using an exosome identification kit. Apoptotic body-related markers, including C1q and Histone H3, were also detected in ABs and DABs by Western blotting.

### Cellular uptake of DABs and ABs

HUVECs, HaCaT cells, and RAW264.7 cells were seeded in 12-well plates at a density of 1 × 10^5^ cells/well and cultured overnight. For confocal laser scanning microscopy, ABs and DABs were labeled with DiI. For flow cytometric analysis, ABs and DABs were labeled with DiO. After labeling, the dye-labeled vesicles were thoroughly washed/purified to remove unbound free dye before incubation with recipient cells. The cells were then incubated with DiI-labeled or DiO-labeled ABs/DABs for 4 h. Untreated cells were included as autofluorescence controls. After incubation, the cells were washed three times with cold PBS to remove non-internalized vesicles. For confocal laser scanning microscopy, cells treated with DiI-labeled ABs/DABs were fixed with 4% paraformaldehyde and stained with DAPI. For flow cytometric analysis, cells treated with DiO-labeled ABs/DABs were collected using trypsin, washed with PBS, resuspended in PBS, and analyzed by flow cytometry.

### Preparation and characterization of Exo

MSCs were cultured in HPL-free DMEM/F12 for 24 h. The conditioned medium was collected and sequentially centrifuged (300×g, 2000×g, and 10000×g), filtered through a 0.22-µm membrane, and ultracentrifuged at 100,000 ×g for 70 min at 4 °C. The exosome pellet was resuspended in PBS and stored at -80 °C. The particle size distribution of exosomes was assessed by dynamic light scattering (DLS), vesicular morphology was visualized using transmission electron microscopy (TEM), and exosome-associated surface markers (CD9, CD63, TSG101, and Hsp70) were analyzed by western blotting. These analyses collectively confirmed the characteristic size distribution, canonical vesicular morphology, and expected marker expression of exosomes (Fig. S17 and S18).

### miRNA sequencing and bioinformatic analysis

Exosomes (Exo) and apoptotic bodies (ABs) were isolated from MSCs, and DFO-programmed apoptotic bodies (DABs) were isolated from DFO-preconditioned MSCs as described above. Small RNA sequencing was performed by Bioyi Biotechnology Co., Ltd. (Wuhan, China). Total RNA was extracted from Exo, ABs, and DABs. Small RNAs of 18 to 30 nucleotides were used to build libraries. These libraries were amplified by PCR and sequenced on the Illumina NovaSeq 6000 platform. Raw reads were filtered for clean data. Differentially expressed miRNAs (DEMs) were identified using thresholds of |fold change| > 2 and p-value < 0.05. Target transcripts were predicted using TargetScan and miRDB. GO and KEGG enrichment analyses were done on these targets. Chord diagrams were made using the Hiplot web tool (http://hiplot.com.cn) to show miRNA-function relationships. Pathway clustering was done using Metascape (https://metascape.org/). Protein-protein interaction (PPI) networks and hub modules were built using Cytoscape with the MCODE plugin. The target genes of the top 10 upregulated miRNAs in DABs were obtained using the multiMiR package by incorporating validated and high-confidence miRNA–target interactions from databases including miRecords and miRTarBase. The complete target-gene list was provided as Supplementary Data 1. These target genes were used for GO biological process enrichment, KEGG pathway enrichment, and PPI network analyses.

### Analysis of HUVEC proliferation

Cell proliferation was measured by CCK-8 assay. HUVEC were seeded in 96-well plates at 5 × 10^3^ cells/well overnight and incubated with Exo, ABs, or DABs (40 µg/mL). Untreated cells served as the control. At 0, 12, 24, and 48 h, culture medium was replaced with fresh medium containing 10% (v/v) CCK-8 reagent and incubated for 1–2 h at 37°C. Absorbance at 450 nm was measured with a microplate reader (Multiskan MK3, Thermo).

### Cell scratch assay

Cell migration was measured by scratch assay. HUVEC and HaCaT cells were seeded in 6-well plates at 2 × 10^5^ cells/well and cultured to confluency. A linear scratch was made with a sterile 200 µL pipette tip, and detached cells were removed by PBS. Cells were cultured in serum-free medium with 40 µg/mL Exo, ABs, or DABs. Images of the scratch area were taken at 0, 12, and 24 h using an inverted phase-contrast microscope, and gap areas were measured with ImageJ.

### Transwell assay

HUVEC suspensions (2 × 10^4^ cells in 200 µL) were seeded in the upper chambers of transwell inserts (24-well, 8 μm pore size). The lower chambers contained 600 µL medium with 5% FBS and 40 µg/mL Exo, ABs, DABs, or PBS. Cells were cultured at 37°C for 24 and 48 h. The upper chamber membrane was fixed with 4% paraformaldehyde and stained with 0.1% crystal violet. Migrated cells were imaged at 24 and 48 h by an inverted optical microscope and counted with ImageJ.

### Matrigel tube formation assay

Angiogenesis was assessed by tube formation assay. HUVEC (5 × 10^4^ cells/well) were seeded in 96-well plates coated with 100 µL growth factor-reduced Matrigel (Corning, USA) and incubated with 40 µg/mL Exo, ABs, or DABs for 8 h. Tube structures were imaged and quantified with ImageJ.

### Macrophage polarization assay in vitro

To evaluate the immunomodulatory capacity of the vesicles, RAW264.7 murine macrophages were plated in 12-well plates (1.5 × 10^5^ cells/well) and primed with lipopolysaccharide (LPS, 1 µg/mL) for 24 h to induce an inflammatory phenotype. The LPS-challenged cells were then cultured for an additional 24 h in fresh complete medium containing PBS or 40 µg/mL of Exo, ABs, or DABs. For flow cytometric analysis, the treated macrophages were harvested, resuspended in 100 µL of PBS, and dual-stained with anti-mouse CD86 and anti-mouse CD206 antibodies for 30 min at 4°C in the dark. After standard washing procedures, the macrophage polarization states were quantified using a flow cytometer.

### Macrophage immunoregulatory assay in vitro

For an in vitro immunoregulatory study, RAW264.7 cells were treated with LPS (1 µg/ml) for 24 h and subsequently co-incubated with different formulations (40 µg/mL). Twenty-four hours later, the levels of immune factors (TNF-α, IL-6, IL-1β, IL-4, IL-10, and VEGF-A) in cell supernatants were measured using ELISA kits according to the manufacturer’s recommended protocols.

### Macrophage ATP production assay in vitro

To evaluate the total adenosine 5’-triphosphate (ATP) production capacity of macrophages in vitro, RAW264.7 cells were seeded in each well of twelve-well plates and treated with Exo, ABs, and DABs (40 µg/mL) for 24 h, and cells in medium containing PBS were prepared in parallel as the control group (1.5 × 10^5^ cells). Afterwards, ATP in macrophages from different groups were extracted and detected using an Enhanced ATP Assay Kit.

### Mitochondrial membrane potential, OCR/ECAR, and ROS detection in macrophages

RAW264.7 cells were treated with PBS, Exo, ABs, or DABs at 40 µg/mL for 24 h under the same conditions used for ATP detection. Mitochondrial membrane potential was evaluated using a JC-1 staining kit according to the manufacturer’s instructions. Briefly, cells were incubated with JC-1 working solution at 37°C for 20 min in the dark, washed with staining buffer, and imaged by fluorescence microscopy. The mitochondrial membrane potential was quantified as the red/green fluorescence intensity ratio using ImageJ.

Mitochondrial respiration and extracellular acidification were assessed by OCR/ECAR analysis. RAW264.7 cells were seeded in the assay plate and treated with PBS, Exo, ABs, or DABs at 40 µg/mL for 24 h. The oxygen consumption rate (OCR) and extracellular acidification rate (ECAR) were measured using an extracellular flux analyzer according to the manufacturer’s protocol. Basal OCR, FCCP-stimulated OCR, and baseline ECAR were quantified to evaluate mitochondrial respiratory activity and extracellular acidification.

Intracellular ROS levels were detected using DCFH-DA staining. After treatment with PBS, Exo, ABs, or DABs for 24 h, cells were incubated with 10 µM DCFH-DA at 37°C for 30 min in the dark, washed with PBS, and imaged by fluorescence microscopy. Fluorescence intensity was quantified using ImageJ.

### Synthesis and characterization of phenylboronic acid-functionalized HA (PHA)

To synthesize the PHA copolymer, 3-aminophenylboronic acid (3-APBA) was grafted onto the hyaluronic acid (HA) backbone via EDC/NHS-mediated amide coupling. First, the carboxyl groups of HA (80 mL, 7.5 mg/mL) were activated by stirring with EDC (540 mg) and NHS (330 mg) at room temperature for 6 h. Following activation, 3-APBA (450 mg) was introduced, and the conjugation reaction proceeded under vigorous stirring for an additional 24 h. To purify the synthesized PHA, the crude mixture was dialyzed (MWCO 3500 Da) against distilled water for 48 h, passed through a 0.8-µm filter, and lyophilized at -80°C. The chemical structure and successful grafting of the final PHA product were subsequently validated utilizing UV-vis spectrophotometry, ^1^H NMR, and FTIR spectroscopy.

### Preparation of the glucose/ROS dual-responsive PHA-PVA^Gel^ hydrogel

To fabricate the PHA-PVA^Gel^ hydrogels, lyophilized PHA (at predetermined concentrations) and commercial PVA were individually dissolved in deionized water, with the pH of both precursor solutions subsequently neutralized to 7.4. The final hydrogel networks were rapidly formed at ambient temperature by co-extruding the PHA and PVA solutions at a 1:1 (v/v) ratio utilizing a double-barrel syringe. Five hydrogel compositions were fabricated with varying PHA solutions: Gel-1 (PHA (1.0%, w/v), PVA (8.0%, w/v)), Gel-2 (PHA (2.0%, w/v), PVA (8.0%, w/v)), Gel-3 (PHA (3.0%, w/v), PVA (8.0%, w/v)), Gel-4 (PHA (4.0%, w/v), PVA (8.0%, w/v)), and Gel-5 (PHA (5.0%, w/v), PVA (8.0%, w/v)).

### Microstructure characterization

The internal structure of PHA-PVA^Gel^ specimens was examined by scanning electron microscopy (SEM). Specimens were snap-frozen in liquid nitrogen and freeze-dried for 24 h. Cross-sections were mounted on conductive carbon stubs, sputter-coated with gold, and imaged by SEM.

### Rheological behavior test

Viscoelastic properties of the hydrogels were measured on a DHR-10 rheometer (TA Instruments) with a 10-mm parallel plate geometry at a 1-mm gap. Time sweeps at 1% strain were first performed to establish the linear viscoelastic region. Frequency sweeps (0.1–100 rad/s, 1% strain) and strain sweeps (0.1–1000%) were then performed at 37°C to measure storage modulus (G’) and loss modulus (G’’).

### Self-healing properties evaluation

Self-healing of PHA-PVA^Gel^ was assessed visually and rheologically. Two hydrogel pieces dyed with rhodamine B and methylene blue were placed in contact for 2 h and then stretched to confirm bonding at the interface. Rheological recovery was measured by a step-strain test, where strain was alternated between 1000% and 5% to monitor recovery of G’ and G’’.

### Adhesion strength test

The in vitro adhesion of the PHA-PVA^Gel^ was assessed on eight distinct organs (heart, liver, lung, spleen, kidney, bladder, colon, and stomach) that were excised from C57 mice. Specifically, 200 µL of freshly prepared PHA-PVA^Gel^ was applied to stainless steel tweezers, which were subsequently used to attach the gel to the moist surface of the organs above. All these tests were conducted more than 5 times to ensure the results were consistent and reliable.

### Intracellular ROS elimination and cytoprotective effect of PHA-PVA^Gel^

To evaluate the intracellular ROS-scavenging capability of the hydrogels, HUVECs (2 × 10^5^ cells/dish) were seeded into confocal imaging dishes and allowed to attach overnight. The cultures were pre-treated with medium containing either HA-PVA^Gel^ or PHA-PVA^Gel^ for 2 h, followed by a 4-h oxidative challenge using 5 mM H_2_O_2_. Intracellular ROS levels were subsequently probed by incubating the cells with 10 µM DCFH-DA (in serum-free medium) for 30 min prior to fluorescence microscopy. HUVEC (1 × 10^5^ cells/well) were seeded in 12-well plates, pre-incubated with hydrogels for 2 h, and treated with 5 mM H_2_O_2_ for 6 h. Cell viability was assessed by LIVE/DEAD^®^ staining and fluorescence imaging. Apoptosis was measured by flow cytometry.

### Evaluation of biocompatibility

Cytotoxicity of PHA-PVA^Gel^ was measured by CCK-8 assay in HUVEC and HaCaT cells. Hydrogel extracts were prepared following ISO 10993-5. To generate the hydrogel leachates for in vitro assays, the mixture was incubated at 37°C for 48 h. For biocompatibility evaluation, HUVECs and HaCaT cells were plated in 96-well plates at a density of 5 × 10^3^ cells/well and allowed to adhere overnight. The standard culture medium was subsequently aspirated and substituted with 200 µL of fresh medium supplemented with the prepared hydrogel leachates. Cellular viability was continuously monitored over a 72-h period (evaluated at 24, 48, and 72 h) utilizing a CCK-8 assay.

### Preparation and characterization of DABs@PHA-PVA^Gel^

The DABs@PHA-PVA^Gel^ hydrogel was prepared through premixing and in situ gelation. Briefly, DABs were isolated as described above and dispersed in the PVA precursor solution at a concentration of 200 µg/mL. The DABs-containing PVA solution was gently pipetted to ensure homogeneous vesicle dispersion before gelation. Subsequently, the PVA solution containing DABs was mixed with the PHA solution using a dual-channel injector at a 1:1 volume ratio to form the dynamic boronate ester-crosslinked PHA-PVA^Gel^ network. During this in situ gelation process, DABs were physically entrapped within the hydrogel matrix rather than covalently conjugated to the polymer backbone. To visualize the spatial distribution of DABs in the hydrogel, DiI-labeled DABs were incorporated into PHA-PVA^Gel^ using the same procedure. Z-stack confocal laser scanning microscopy was then performed to evaluate the three-dimensional distribution of DABs within the hydrogel network.

### In vitro responsive particle release assay

To simulate the microenvironment of diabetic wounds, three release media were prepared: (1) H_2_O_2_ solution (200 µM), (2) glucose solution (16 mM), and (3) H_2_O_2_ (200 µM) and glucose (16 mM) mixed solution. Then, the release profile of DABs from DABs@PHA-PVA^Gel^ was examined when submerged in PBS and the medium at 37°C. Briefly, DABs@PHA-PVA^Gel^ was positioned within 12-well plates, with each well containing 2 mL of the corresponding release medium, ensuring complete immersion of the hydrogel. The plates were subsequently incubated in a shaking incubator at 37°C with constant agitation. At predetermined time intervals (0, 2, 4, 6, 8, 12, 24, 36, 48, 60, and 72 h), 400 µL of the release medium was carefully collected from each well, and an equivalent volume of fresh medium was introduced to preserve a consistent volume of 2 mL. The number of DABs released at predetermined intervals was determined via BCA protein assay. In addition, the size distribution of DABs in the liquid media at 72 h was measured using a laser particle size meter to assess shape integrity and changes in diameter after release.

To evaluate whether hydrogel encapsulation and release affected DABs integrity, DABs released from PHA-PVA^Gel^ were collected and compared with initial DABs before hydrogel encapsulation. Briefly, DABs@PHA-PVA^Gel^ was incubated in the glucose/ROS-mimicking release medium at 37°C for 72 h, corresponding to the dressing-change interval used in the in vivo wound-healing experiment. The released DABs were collected from the supernatant and concentrated for subsequent characterization. The morphology of initial and released DABs was observed by transmission electron microscopy. Phosphatidylserine exposure was evaluated by Annexin V-FITC staining and flow cytometry. Apoptotic body-related markers, including C1q and Histone H3, were analyzed by Western blotting. To evaluate post-release bioactivity, released DABs and initial DABs were normalized by total protein concentration. HUVEC proliferation was assessed using the CCK-8 assay under the same conditions described above. In addition, RAW264.7 macrophages were incubated with initial DABs or released DABs, and intracellular ATP production was measured using an ATP assay kit according to the manufacturer’s instructions.

Preliminary storage stability and batch-to-batch reproducibility of DABs were evaluated to assess formulation robustness. For storage stability, freshly prepared DABs were stored at − 20 °C–− 80 °C for 30 days, and their hydrodynamic size was measured at the indicated time points by DLS. For batch-to-batch reproducibility, three independent DABs batches were prepared from the same parental MSC input under identical conditions. DABs yield was quantified by BCA protein assay and normalized to the parental MSC input. To compare batch-to-batch bioactivity, DABs from the three batches were incubated with HUVECs at the same protein concentration, and HUVEC proliferation was evaluated by CCK-8 assay.

### In vitro responsive degradation test

The degradation assay was conducted by immersing the DABs@PHA-PVA^Gel^ in the three previously mentioned microenvironmental media. In short, DABs@PHA-PVA^Gel^ was freshly prepared, and the surface moisture was removed using filter paper. The initial weight (W_0_) was then recorded. Subsequently, the weighed samples were incubated in the above-mentioned media and PBS at 37 °C. At designated time points (3, 6, 9, 12, and 15 days), the medium was discarded, and the hydrogels were dried and weighed (W_n_). The degradation rate of the hydrogels was calculated using the following formula: DR = (W_0_ - W_n_) / W_0_ × 100%, where W_0_ represents the initial weight of the hydrogel at day 0, W_n_ denotes the weight of the hydrogel at day n, and DR indicates the degradation rate of the hydrogel.

### Establishment of diabetic mouse cutaneous wound model

All in vivo protocols were executed under the ethical approval of the Institutional Animal Care and Use Committee (IACUC) at China Pharmaceutical University (approval # 2024-09-017). Male C57BL/6 mice (6 weeks old, 18–22 g) were sourced from Jiangsu Huachuang Sino Pharmaceutical Technology Co., Ltd. and maintained on a 12-h light/dark cycle with ad libitum access to a high-fat diet and water. To induce the type 2 diabetes mellitus (T2DM) model, mice reaching a body weight of 30 g received consecutive daily intraperitoneal injections of streptozotocin (STZ, 50 mg/kg) for 5 days. Fasting blood glucose (FBG) was measured by a glucometer. Mice with FBG > 16.65 mM for two consecutive days were considered successfully modeled. Mice that did not reach this threshold received additional STZ (20 mg/kg, i.p.). Diabetic mice were anesthetized, dorsal hair was removed, and a non-splinted 10-mm full-thickness excisional wound was made along the dorsal midline.

### In vivo wound healing investigation

The diabetic mice were randomly divided into 8 groups: (1) Control group (G1); (2) Exo group (G2); (3) ABs group (G3); (4) DABs group (G4); (5) PHA-PVA^Gel^ group (G5); (6) ABs@PHA-PVA^Gel^ group (G6); (7) DABs@usGel group (G7); and (8) DABs@PHA-PVA^Gel^ group (G8). (Herein, ABs@PHA-PVA^Gel^ denotes a responsive hydrogel system for ABs, whereas DABs@usGel represents an unresponsive hydrogel loaded with DABs, prepared through freeze-thaw crosslinking using HA and PVA as precursor solutions at the same concentration.) Each group contained at least six mice. Treatments were administered every 3 days according to the formulation type. For the free vesicle groups, freshly prepared Exo, ABs, or DABs suspensions were topically applied to the wound surface. For the hydrogel groups, the residual hydrogel dressing was gently removed, and a freshly prepared hydrogel formulation was applied to cover the wound area. No additional intradermal or subcutaneous injection of DABs was performed during routine treatment. The 3-day treatment interval was selected according to the in vitro release profile of DABs@PHA-PVA^Gel^ and corresponded to the dressing-change interval used in this study. All procedures were performed under aseptic conditions to minimize wound contamination. Exo, ABs, and DABs were dosed at 100 µg/mouse, and the control group received saline. Wounds were covered with 3 M™ Tegaderm dressings. Mice were euthanized at day 7 and day 14, and full-thickness dorsal skin was collected for further analysis.

Macroscopic wound closure was monitored by capturing digital photographs on days 0, 3, 7, and 14 post-surgeries. Additionally, the wound area ratio (%) can be calculated by the formula S_n_/S_0_ × 100%, where S_0_ is the wound area at day 0, and S_n_ is the wound area at day n (*n* = 3, 7, 14). On day 7 and 14, the mice were anesthetized and euthanized, after which the wound tissues were harvested for histological analysis.

### Histological, immunohistochemistry, and immunofluorescence analysis

For histological evaluation, tissue sections were deparaffinized and rehydrated, then stained with H&E and Masson’s trichrome. To evaluate the expression of ROS in the wounds of diabetic mice, immunofluorescence staining was performed to examine ROS levels in the skin tissues of the wound areas during the intermediate treatment phase (day 7). To assess macrophage phenotypic transition in wounds, immunofluorescence staining for CD86 and CD206 was performed. To detect angiogenesis in the wound, wound tissue sections were stained with CD31, CD105, and α-SMA for immunofluorescence. In addition, IHC staining for VEGF-A and TGF-β was performed to detect changes in the wound microenvironment associated with neovascularization. To evaluate hypoxia-induced improvement in mouse wounds, immunofluorescence staining was performed to assess HIF-1α expression. Nuclei were stained with DAPI. Quantification of IHC and IF was performed using ImageJ software.

### Evaluation of tissue inflammation

At days 7 and 14, tissue sections were collected for the assessment of tissue inflammation. To quantify TNF-α, IL-6, IL-1β, IL-4, IL-10, and VEGF-A levels, cytokine data were standardized relative to the concentrations of these factors measured in normal skin using ELISA kits. Furthermore, flow cytometry was used to analyze macrophage polarization in skin wound tissues at the conclusion of the treatment period.

### Transcriptome analysis

Wound tissues from the control and DABs@PHA-PVA^Gel^ groups (*n* = 3/group) were collected at day 14. Total RNA was extracted with TRIzol (Invitrogen) and quantified by a NanoDrop 2000c spectrophotometer (Thermo Fisher Scientific). Libraries were constructed from high-quality RNA and sequenced on the BGISEQ-T7 platform (BGI, Shenzhen, China). Raw FASTQ reads were filtered with fastp (v0.21.0) and aligned to the mouse reference genome with HISAT2 (v2.1.0). Transcript expression was quantified as FPKM using StringTie (v2.1.5). DEGs were identified with DESeq2 using thresholds of |log2(fold change)| > 1 and *p* < 0.05. GO and KEGG enrichment analyses were performed with the clusterProfiler R package (*p* < 0.05).

### qPCR and Western blot validation of RNA-seq-identified pathways

To validate key pathways identified by RNA-seq, wound tissues from the untreated and DABs@PHA-PVA^Gel^ groups were collected for qPCR and Western blot analysis. Total RNA was extracted from wound tissues, reverse-transcribed into cDNA, and subjected to qPCR analysis for Txnip, Foxo4, Daglb, Cnr2, Cxcr4, Nrp1, and S1pr1. Relative mRNA expression was calculated using the 2^−ΔΔCt^ method. The primer sequences used for RT-qPCR are listed in Tab. S3.

For Western blot analysis, total proteins were extracted from wound tissues using RIPA buffer. Equal amounts of protein were separated by SDS-PAGE and transferred onto PVDF membranes. After blocking, membranes were incubated with primary antibodies against Txnip, Foxo4, Daglb, Cnr2, Cxcr4, Nrp1, S1pr1, and Gapdh, followed by incubation with HRP-conjugated secondary antibodies. Protein bands were visualized using enhanced chemiluminescence, and relative protein expression was quantified using ImageJ.

### Statistical analysis

Statistical analysis was performed using GraphPad Prism 10.4.1. Data are presented as mean ± SD. Two-group comparisons were made by two-tailed unpaired Student’s t-test. Multi-group comparisons were made by one-way ANOVA with Tukey’s multiple comparisons. *P* < 0.05,* p < 0.01, and p < 0.001* were considered statistically significant.

## Results

### Preparation and characterization of DABs

**Fig. 2:**
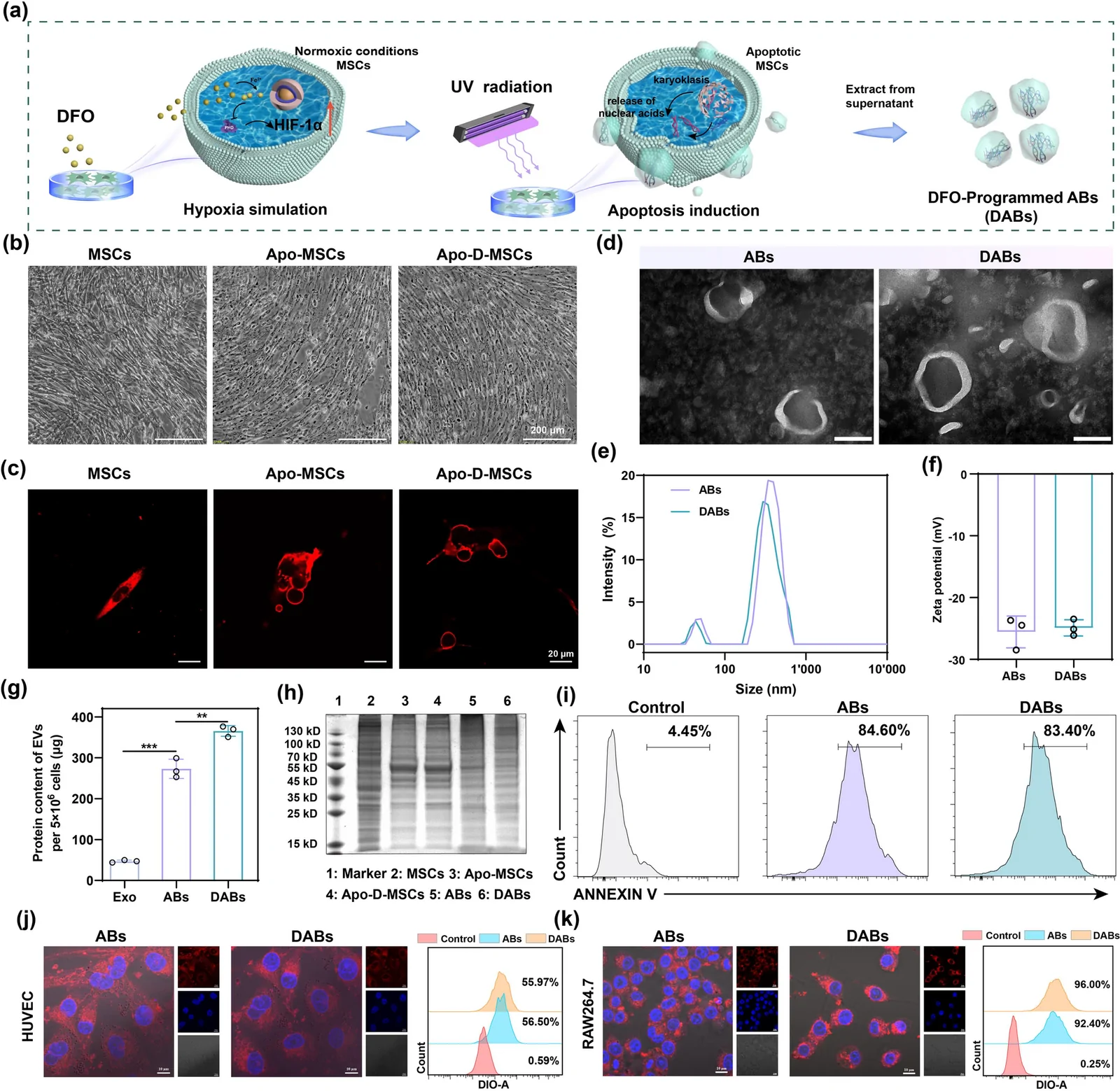
Preparation and characterization of ABs and DABs. (**a**) Schematic illustration of the isolation and purification of DABs from DFO-treated MSCs. (**b**) Morphological changes of MSCs 6 h after apoptosis. (Scale bars = 200 μm). (**c**) Confocal images showing DiI-labeled apoptotic MSCs with membrane blebbing and apoptotic vacuole-like structures at 6 h after apoptosis induction. Scale bar = 20 μm. (**d**) TEM of ABs and DABs (Scale bars = 100 nm). (**e**) Particle size distribution of ABs and DABs. (**f**) Zeta potential of ABs and DABs. (**g**) Relative protein content in Exo, ABs, and DABs (*n* = 3). (**h**) Protein composition of MSCs, Apo-MSCs, Apo-D-MSCs, ABs, and DABs was analyzed by Coomassie blue staining. (**i**) Flow cytometric analysis of Annexin V staining in ABs and DABs. (j) & (**k**) CLSM images were obtained using DiI-labeled ABs/DABs, whereas flow cytometry was performed using DiO-labeled ABs/DABs. (Scale bar = 10 μm). **p < 0.01, ***p < 0.001

The preparation of DABs is shown in Fig. 2a. Before DABs generation, the immunophenotype of MSCs was further verified by flow cytometry. MSCs were negative for CD45 and HLA-DR, while showing high expression of CD73 and CD105, supporting their mesenchymal phenotype (Fig. S1). MSCs were preconditioned with DFO and then induced to undergo apoptosis by UV irradiation. To optimize DFO preconditioning, MSCs were treated with various concentrations of DFO. Based on AO/PI cell viability assays and Western blot analysis, 250 µg/mL was selected as the optimal concentration, as it induced the maximum upregulation of HIF-1α while maintaining excellent cell viability (Fig. S2 and Fig. S3). Subsequently, the optimal UV-induced apoptosis condition was determined to be 10 min of irradiation, based on analysis of cell morphology and apoptosis rates (Fig. S4 and Fig. S5). To verify whether DFO-induced HIF-1α stabilization remained detectable after apoptosis induction, HIF-1α expression was further examined after DFO pretreatment followed by UV irradiation and 24 h apoptosis induction under the same conditions used for DABs generation. Western blot analysis showed that HIF-1α expression remained elevated in DFO-pretreated MSCs after UV-induced apoptosis, with the strongest signal observed in the 250 µg/mL group (Fig. S6a and S6b).

Following UV irradiation, the morphology of the apoptotic cells was examined. At six hours post-treatment, both apoptotic MSCs (Apo-MSCs) and apoptotic DFO-MSCs (Apo-D-MSCs) exhibited clear morphological signs of apoptosis, including the formation of prominent vacuole-like structures and membrane blebbing, which were absent in the non-irradiated control group (Fig. 2b and c). Following 24 h of induction, apoptotic bodies (ABs and DABs) were isolated through differential sequential centrifugation (Fig. S7). Transmission electron microscopy (TEM) revealed that both vesicle types displayed spherical or cup-shaped membrane-bound morphology (Fig. 2d). Dynamic light scattering (DLS) analysis showed a broad hydrodynamic size distribution of approximately 50–800 nm for ABs and DABs in PBS (Fig. 2e). The apparent difference between TEM and DLS measurements is mainly attributable to their different detection principles. DLS measures the hydrodynamic diameter of vesicles in suspension and is sensitive to larger vesicles or minor aggregation, whereas TEM visualizes dried and stained vesicles and may underestimate vesicle size due to sample dehydration. Therefore, DLS was used to evaluate the overall hydrodynamic size distribution, while TEM was used to confirm membrane-bound vesicular morphology. Both ABs and DABs displayed a negative surface charge of approximately − 20 mV, consistent with their origin from the negatively charged cell membrane (Fig. 2f).

We then examined the key surface properties of the vesicles. Coomassie brilliant blue staining verified that both ABs and DABs contained a complex protein from their parent cells (Fig. 2h). Both ABs and DABs were found to be highly enriched with the “eat-me” signal, phosphatidylserine (PS), with flow cytometry showing exposure rates of 84.60% and 83.40%, respectively (Fig. 2i). To further evaluate the apoptotic body identity and exosomal signature of the isolated ABs and DABs, we compared Annexin V-positive PS exposure among Exo, ABs, and DABs. Exo showed minimal Annexin V-positive PS signal, whereas ABs and DABs exhibited markedly higher PS exposure, with Annexin V-positive rates of 85.8% and 88.9%, respectively, supporting the apoptotic body characteristics of ABs and DABs with minimal exosomal signature (Fig. S8). In addition to Annexin V-based PS detection, Western blot analysis showed that the apoptotic body-related markers C1q and Histone H3 were clearly detected in both ABs and DABs (Fig. S9). We further compared exosome-associated markers among Exo, ABs, and DABs. CD63 and CD9 were enriched in Exo but were weak or nearly undetectable in ABs and DABs, whereas Calnexin was clearly detected in ABs and DABs but minimal in Exo (Fig. S10). These results, together with the broad hydrodynamic size distribution, membrane-bound morphology, and high PS exposure of ABs and DABs, collectively demonstrate that the isolated ABs and DABs were high-purity apoptotic body preparations with minimal exosomal signature. BCA assay showed that DAB yield was higher than ABs yield and Exo yield from the same number of parent cells (Fig. 2g). This result confirmed that DFO-programmed apoptotic body production retained the high-yield advantage of apoptosis-derived vesiculation, while the subsequent small RNA sequencing analysis further demonstrated DFO-induced cargo remodeling.

Afterwards, HUVEC, RAW264.7, and HaCaT cells were incubated with DiI-labeled ABs and DABs. Confocal microscopy showed that all three cell types internalized ABs and DABs with perinuclear localization. Flow cytometry showed that RAW264.7 had higher fluorescence intensity than HUVEC and HaCaT cells, indicating greater uptake efficiency (Fig. 2j and k; Fig. S11, S12, and Fig. S13). This may be due to PS on the surface of ABs and DABs, which promotes recognition and uptake by macrophages via efferocytosis.

### Transcriptomics analysis of DABs

**Fig. 3:**
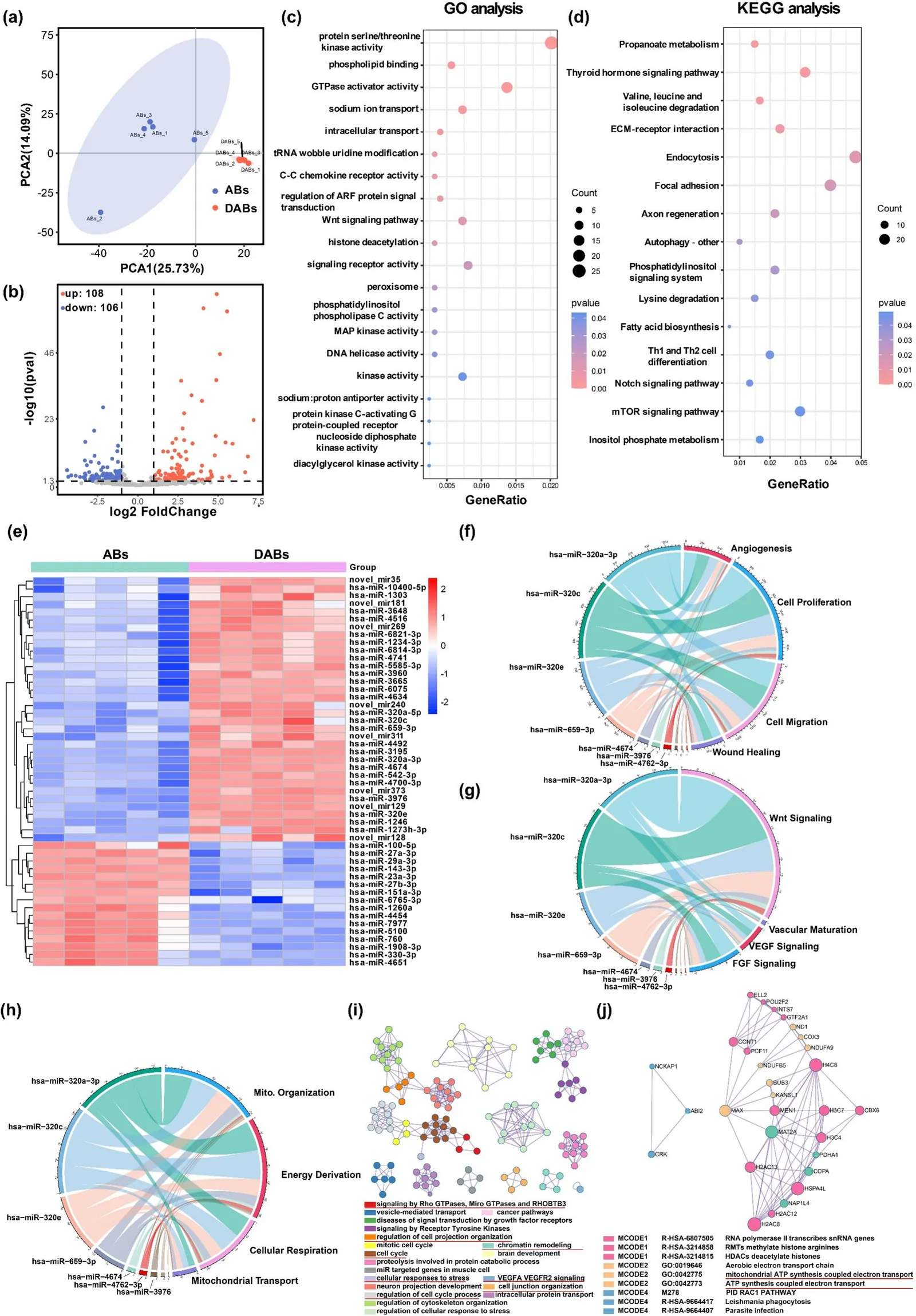
Bioinformatic comparison between DABs and ABs. (**a**) Principal component analysis (PCA) of the transcriptomic profiles between ABs and DABs. Each data point represents a single sample. (**b**) Volcano plots to exhibit the upregulated (108) and downregulated (106) miRNAs (ABs vs. DABs). (**c**) Gene ontology (GO) enrichment and (**d**) Kyoto Encyclopedia of Genes and Genomes (KEGG) pathway enrichment analysis upon 214 differential miRNAs. (**e**) Heatmap showing the significantly differential miRNA (top 50) expression between ABs and DABs (*p* < 0.05, FDR < 0.05). (**f**) Chord plot illustrating the functional associations between the top 10 upregulated miRNAs in DABs and key regenerative biological processes. (**g**) Chord plot showing the relationships between the top 10 upregulated miRNAs in DABs and representative angiogenesis-related signaling pathways, including Wnt signaling, VEGF signaling, FGF signaling, and vascular maturation. (**h**) Chord plot showing the relationships between the top 10 upregulated miRNAs in DABs and representative mitochondrial processes, including mitochondrial organization, energy derivation, cellular respiration, and mitochondrial transport. (**i**) Metascape functional visualization analysis showing the process and pathway enrichment network (colored by cluster ID) from the target genes of the top 10 upregulated miRNAs in DABs. (**j**) PPI network showing the interaction relationship among the predicted target genes of the top 10 significantly upregulated miRNAs in DABs, with the hub gene networks identified by the MCODE algorithm

miRNAs play key roles in post-transcriptional gene regulation and cell modulation [27, 28]. The miRNA content of vesicles reflects the physiological state of the parent cell [29]. To investigate the impact of DFO programming on vesicular cargo, we compared miRNA profiles of ABs and DABs by high-throughput sequencing. PCA showed clear separation between the two groups, with DABs showing tighter clustering and greater sample homogeneity (Fig. 3a). Differential expression analysis identified 214 altered miRNAs in DABs, including 108 upregulated and 106 downregulated (Fig. 3b).

To elucidate the functional implications of the differentially expressed miRNAs, we performed GO and KEGG enrichment analyses. Then GO and KEGG enrichment analyses were performed on predicted target genes of the differentially expressed miRNAs (DEMs). GO analysis showed enrichment in protein kinase activity and chemokine receptor signaling (Fig. 3c). KEGG analysis showed enrichment in immune-related pathways such as Th1/Th2 differentiation, as well as focal adhesion and axon regeneration pathways (Fig. 3d). Analysis of the top 50 DEMs showed that 34 were upregulated in DABs (Fig. 3e). miRNAs linked to angiogenesis and cell migration, including hsa-miR-4674, hsa-miR-1246, hsa-miR-3976, and hsa-miR-320e, were enriched in DABs (Fig. S14a and S14b).

To understand how DABs drive regeneration, we looked at the target networks of the top ten upregulated miRNAs in DABs. Pathway enrichment showed these targets are linked to key repair processes, including angiogenesis, cell migration, and VEGF signaling (Fig. 3f and g). We also found that these miRNAs appear to regulate mitochondrial bioenergetics and cellular respiration (Fig. 3h), which was somewhat unexpected. This suggests DABs may promote tissue repair by activating angiogenic pathways while also supporting the metabolic demands of the repair process, though the exact interplay between these two mechanisms needs further investigation. Beyond metabolic reprogramming, enrichment analysis of the top 10 upregulated miRNA targets in DABs revealed a significant clustering in pathways essential for cellular dynamics, such as Rho GTPase signaling and cytoskeleton organization (Fig. 3i) [30]. Combined with enrichments in the mitotic cell cycle and chromatin remodeling, these findings underscore a shift toward a more active, migratory phenotype. How DFO programming integrates these diverse structural and bioenergetic processes remains an open question. To improve the transparency of the miRNA target prediction analysis, the complete predicted target-gene list for the top 10 upregulated miRNAs in DABs is provided as Supplementary Data 1. These targets were obtained using the multiMiR package by incorporating validated and high-confidence miRNA–target interactions from miRecords and miRTarBase. GO and KEGG enrichment analyses based on these predicted target genes further supported the involvement of DAB-enriched miRNAs in chromatin remodeling, epigenetic regulation, oxidative phosphorylation, FoxO signaling, apoptosis, organelle organization, and metabolic regulation (Fig. S15).

Mapping the predicted miRNA targets onto a protein-protein interaction network revealed a rather heterogeneous regulatory landscape, with MCODE identifying two functionally distinct hubs (Fig. 3j, Fig. S16) [30]. A prominent energy-metabolism module (MCODE 2) captures much of the mitochondrial ATP synthesis and electron transport machinery—specifically components like NDUFB5, NDUFA9, COX3, and ND1, which appear tightly governed by the miR-320 family and miR-3976 [31]. The second was a cytoskeletal dynamics module (MCODE 4) centered on RAC1 signaling, where CRK, NCKAP1, and ABI2 formed a closely connected motility network. These results suggest that DABs may work by boosting mitochondrial energy production while also activating RAC1-driven cytoskeletal changes, which together could support cell migration and tissue repair though the relative contribution of each arm warrants further study [32, 33].

Finally, we extended the transcriptomic profiling to include parent cell-derived exosomes (Exo) for comparison. The data showed that both ABs and DABs possessed a more complex and abundant miRNA repertoire compared to the Exo group (Fig. S19-S25). Chord diagram analysis of the top upregulated miRNAs in DABs relative to Exo unveiled a coordinated bipartite regulatory network that simultaneously orchestrates the resolution of inflammation and the activation of structural regeneration pathways. These data provided a mechanistic basis for the enhanced biological activity of DABs under the tested conditions, particularly from the perspective of miRNA cargo remodeling.

### In vitro biological evaluation of DABs

**Fig. 4:**
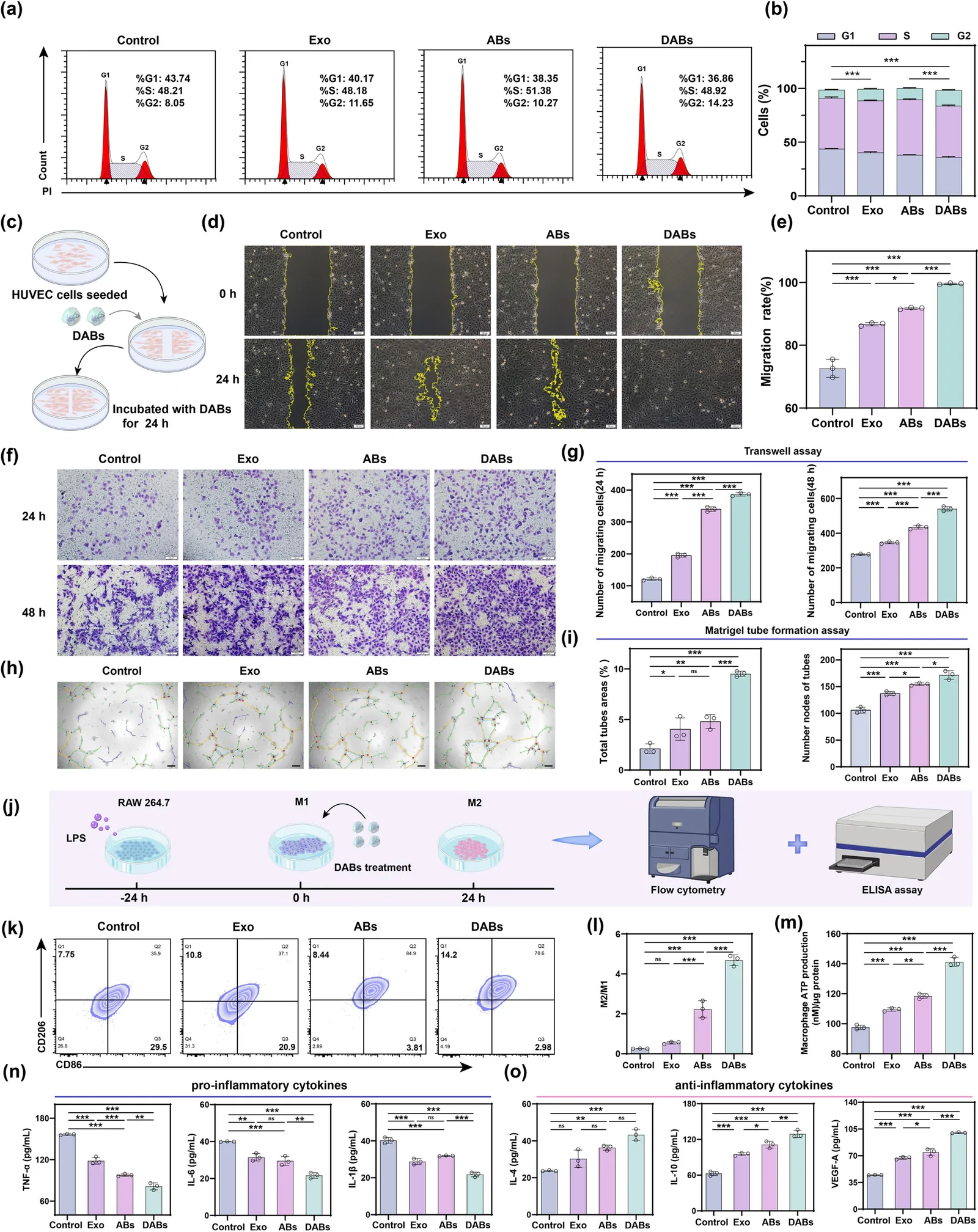
The effects of DABs on cell proliferation, migration, angiogenesis, and anti-inflammation. (**a**) Flow cytometry analysis of HUVEC cells’ cycle distribution and (**b**) corresponding quantitative analysis (n = 3). (**c**) Schematic illustration showing the design of the scratch assay. (**d**) Representative images of scratch assay of HUVEC at 24 h. (e) Quantification of cell migration rate at 24 h (n = 3). (**f**) Representative images of the transwell assay in HUVEC cells treated with Exo, ABs, and DABs, and (**g**) Quantitative analysis of the migrated cells (n = 3). (**h**) Representative images of tube formation assays in HUVEC treated with Exo, ABs, and DABs. (Scale bar = 100 µm) (**i**) Quantitative analysis of the tube formation assays in HUVEC treated with Exo, ABs, and DABs (n = 3). (**j**) Schematic illustration of the in vitro immunomodulation assay using LPS-stimulated RAW264.7. (**k**) Representative flow cytometry plots showing the population shift from pro-inflammatory M1 to anti-inflammatory M2 phenotypes following different treatments. (**l**) Quantitative analysis of the M2/M1 polarization ratio (CD206^+^/CD86^+^) (n = 3). (**m**) Effects of Exo, ABs, and DABs on the total adenosine 5’-triphosphate (ATP) production in RAW264.7 in vitro (*n* = 3). (**n**) Detection of pro-inflammatory cytokines (TNF-α, IL-6, and IL-1β) and (**o**) anti-inflammatory cytokines (IL-4, IL-10, and VEGF-A) in the supernatant of LPS-stimulated RAW264.7 treated with Exo, ABs, and DABs (*n* = 3). *p < 0.05, **p < 0.01, ***p < 0.001

Determining the functional impact of this molecular became our primary focus, particularly given the distinct pro-regenerative miRNA signature we found packaged within the DABs. To provide the therapeutic benchmark, we selected stem cell-derived exosomes (Exo), currently under investigation in clinical trials, as the positive control [34, 35]. Given that effective wound healing necessitates the coordinated proliferation and migration of endothelial and epidermal cells [36], we systematically evaluated the efficacy of DABs against native ABs and Exo in driving these cellular behaviors. DABs visibly forced a rapid G1-to-S transition in HUVECs, pooling the population in the active S and G2/M phases (Fig. 4a and b). Far from just dividing faster, these cells became highly mobile. In fact, 24 h was all it took for DAB-treated HUVEC and HaCaT cultures to completely seal scratch wounds, leaving ABs and Exo lagging far behind (Fig. 4c-e; Fig. S26a and 26b). CCK-8 assays also showed increased cell proliferation after DABs treatment (Fig. S27). Transwell assays showed DABs increased HUVEC migration by 1.95-fold, compared to 1.25-fold with Exo and 1.16-fold with native ABs (Fig. 4f and g). In Matrigel tube formation assays, DABs also produced the greatest increases in total tube length and network area across all groups (Fig. 4h and i).

To evaluate immunomodulatory effects in vitro, RAW264.7 were stimulated with LPS to mimic an inflammatory environment (Fig. 4j) [37]. Flow cytometry showed that the control and Exo groups maintained a high proportion of CD86^+^ (M1) macrophages, while DABs treatment shifted the balance toward CD206^+^ (M2) macrophages (Fig. 4k). The CD206/CD86 ratio was highest in the DABs group compared to both Exo and native ABs (Fig. 4l). ELISA results further showed that DABs treatment lowered pro-inflammatory cytokines (TNF-α, IL-6, and IL-1β) (Fig. 4n) and raised anti-inflammatory and angiogenic cytokines, including IL-4, IL-10, and VEGF-A (Fig. 4o). Polarization into an M2 state fundamentally relies on oxidative phosphorylation. Tracking this specific energy requirement showed a distinct metabolic difference, with DABs-treated macrophages producing substantially more ATP (Fig. 4m). To further substantiate this mitochondrial phenotype, we performed JC-1 staining, OCR/ECAR analysis, and intracellular ROS detection in RAW264.7 cells under the same treatment conditions used for ATP detection. JC-1 staining showed that DABs treatment increased the red/green fluorescence ratio compared with Exo and ABs, indicating improved mitochondrial membrane potential (Fig. S28). OCR analysis further revealed that DABs markedly increased both basal oxygen consumption and FCCP-stimulated oxygen consumption, suggesting enhanced mitochondrial respiratory capacity (Fig. S29a and S29b). In contrast, ECAR was significantly reduced in the DABs group, indicating decreased extracellular acidification and a relative shift toward mitochondrial oxidative metabolism (Fig. S29c). ROS staining also showed that DABs reduced intracellular ROS accumulation in macrophages (Fig. S30). Together, these results provide additional evidence that DABs improve mitochondrial functional status and oxidative metabolic remodeling in recipient macrophages, which may help support the energetic demands of macrophage phenotypic regulation.

### Preparation and characterization of DABs@PHA-PVA^Gel^

**Fig. 5:**
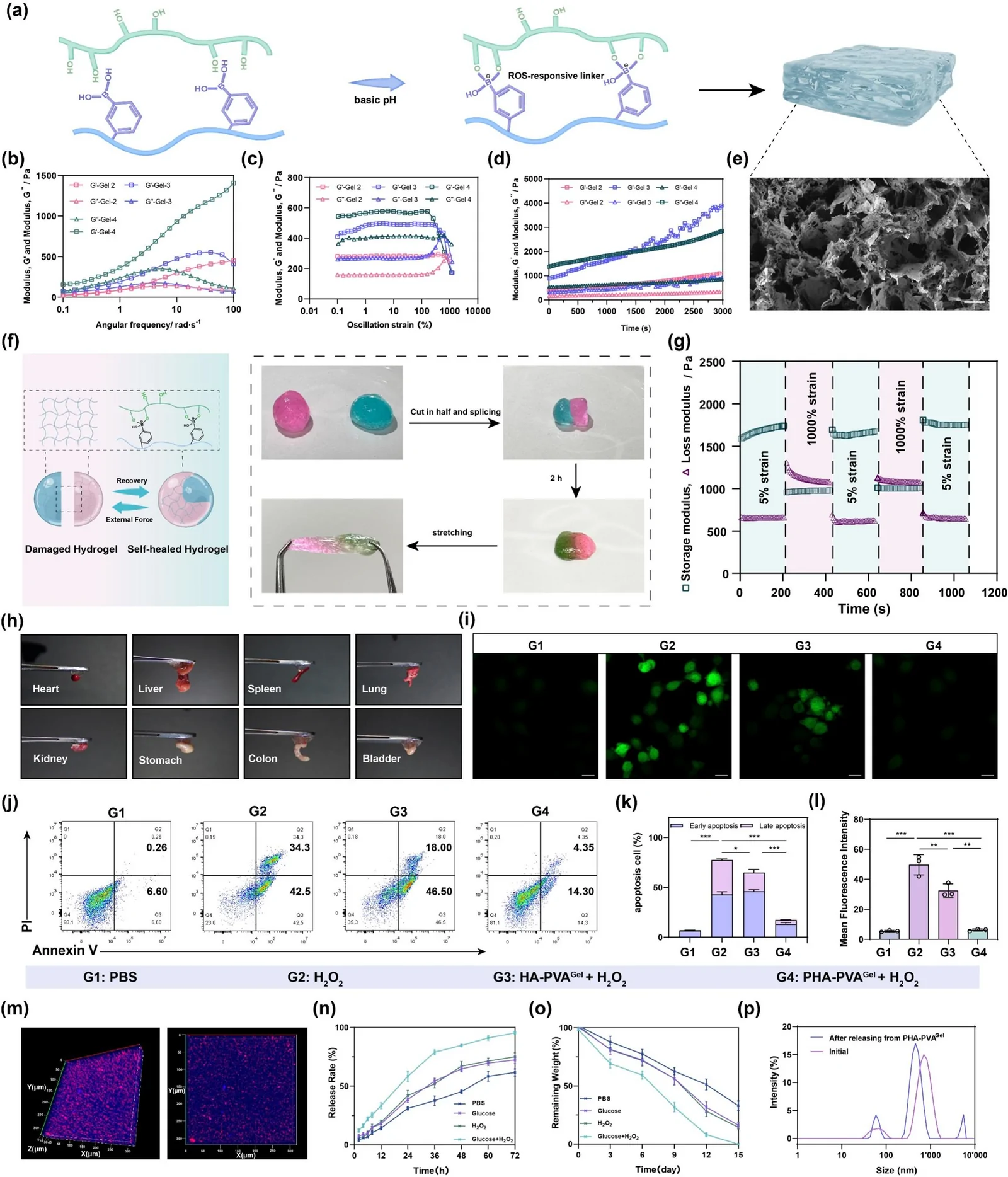
Preparation and characterization of DABs-loaded dual-responsive hydrogel (DABs@PHA-PVA^Gel^). (**a**) Schematic illustration showing the fabrication process of PHA-PVA^Gel^. (**b**) Frequency sweeps tests of PHA-PVA^Gel^. (**c**) Amplitude sweeps tests of PHA-PVA^Gel^. (**d**) Time sweeps tests of PHA-PVA^Gel^. (**e**) SEM characterizes the morphology of PHA-PVA^Gel^. (Scale bar = 100 μm). (**f**) Schematic diagram of PHA-PVA^Gel^ self-healing function and Macroscopic self-healing property of PHA-PVA^Gel^ over time. (**g**) Microscopic self-healing performance of PHA-PVA^Gel^ with stress variation. (**h**) The adhesive effect of PHA-PVA^Gel^ on major organs of C57 mice. (**i**) Representative ROS staining (green fluorescence) of HUVEC with DCFH-DA probe. H_2_O_2_, 1 mM. Scale bar = 20 μm. (**j**) Quantification of ROS fluorescence (*n* = 3). (**k**) Flow cytometry analysis of apoptosis in HUVEC cells co-cultured with PHA-PVA^Gel^ under high oxidative stress conditions. (**l**) Quantitative analysis of HUVEC apoptosis cells after different treatments (*n* = 3). (**m**) Three-dimensional confocal laser scanning microscopy image of DiI-labeled DABs incorporated within PHA-PVA^Gel^. (**n**) DABs release features in different medium (*n* = 3). (**o**) In vitro degradation feature of DABs@PHA-PVA^Gel^ (*n* = 3). (**p**) Particle size of DABs distribution after releasing from DABs@ PHA-PVA^Gel^. (*p < 0.05, **p < 0.01, ***p < 0.001.)

To enable controlled delivery of DABs, we developed a responsive hydrogel using a phenylboronic acid-modified hyaluronic acid copolymer (PHA). PHA was synthesized by conjugating 3-aminophenylboronic acid onto the hyaluronic acid backbone, and successful grafting was confirmed by UV-vis, ^1^H-NMR, and FT-IR spectroscopy (Fig. S31-S33). The copolymer was then crosslinked with polyvinyl alcohol (PVA) to form the hydrogel network (Fig. 5a). Different PHA/PVA ratios were tested to optimize gelation and stability, and vial inversion tests identified Gel-2, Gel-3, and Gel-4 as suitable candidates based on their rapid sol-gel transition and stable formation (Fig. S34).

Rheological characterizations showed that all three formulations were elastic-dominant (G’ > G’’) (Fig. 5b and c). However, Gel-3 displayed fluctuations in its time-sweep profile, suggesting network inhomogeneity (Fig. 5d). Gel-4 maintained a consistent rheological profile and was selected for subsequent experiments (designated as PHA-PVA^Gel^). Scanning electron microscopy (SEM) of PHA-PVA^Gel^ revealed a porous, interconnected microstructure (Fig. S35 and Fig. 5e).

Macroscopic evaluation of self-healing properties showed that two PHA-PVA^Gel^ pieces (stained red and blue) fused within minutes and withstood subsequent stretching (Fig. 5f). Rheological step-strain tests indicated that the hydrogel recovered over 95% of its initial storage modulus immediately following a 1000% high-strain cycle (Fig. 5g). The hydrogel also adhered to various murine organs ex vivo, including the heart, liver, spleen, and lungs (Fig. 5h).

We evaluated the intrinsic ROS-scavenging capacity of the hydrogel in vitro using an H_2_O_2_-induced oxidative stress model in HUVEC. DCFH-DA staining indicated high intracellular ROS levels in the H_2_O_2_-treated group (G2), which were effectively neutralized in the presence of PHA-PVA^Gel^ (G4) (Fig. 5i and l). Flow cytometry showed that H_2_O_2_ induced apoptosis in 76.80% of cells (G2). Treatment with PHA-PVA^Gel^ reduced the apoptotic rate to 18.65% (G4), compared to 64.50% with the control hydrogel (G3) (Fig. 5j and k). Live/Dead staining confirmed higher cell viability in the PHA-PVA^Gel^ group (Fig. S36). Quantitative assays demonstrated that cells cultured in hydrogel leachates-maintained proliferation rates comparable to untreated controls (Fig. S37).

DABs were then incorporated into PHA-PVA^Gel^ through premixing with the PVA precursor solution followed by in situ dynamic boronate ester crosslinking with PHA, yielding DABs@PHA-PVA^Gel^ for further characterization. To visualize DABs distribution within the hydrogel matrix, DiI-labeled DABs were incorporated using the same procedure and analyzed by Z-stack confocal microscopy. The three-dimensional reconstruction and XY projection images showed that DiI-labeled DABs were distributed throughout the hydrogel matrix (Fig. 5m). Furthermore, higher-magnification orthogonal confocal images clearly showed DABs fluorescence signals along the Z-axis, demonstrating successful spatial incorporation and physical entrapment of DABs within the hydrogel network (Fig. S38). We evaluated the in vitro release of DABs under conditions simulating the high glucose and ROS levels of a diabetic wound [38]. Under dual-stimulus conditions (glucose + H_2_O_2_), DABs release reached 95.33% at 72 h, which was significantly higher than either single-stimulus (glucose or H_2_O_2_ alone) or PBS groups (Fig. 5n). This matched the hydrogel degradation pattern, where the greatest mass loss also occurred under dual-stimulus conditions (Fig. 5o). DLS confirmed that the released DABs maintained their original size distribution (Fig. 5p).

To further determine whether hydrogel encapsulation preserved the structural integrity and apoptotic body-related composition of DABs during a therapeutically relevant release period, DABs released from PHA-PVA^Gel^ were collected after incubation in the glucose/ROS-mimicking release medium for 72 h. TEM images showed that released DABs retained membrane-bound vesicular morphology like initial DABs, indicating that the encapsulation and 72 h release process did not visibly disrupt vesicle structure (Fig. S39). Annexin V-FITC analysis further showed that released DABs maintained high PS exposure, with an Annexin V-positive rate of 80.3%, comparable to that of initial DABs (85.4%) (Fig. S40). Western blot analysis showed that C1q and Histone H3 were still clearly detected in released DABs (Fig. S41). Functionally, released DABs retained pro-proliferative activity in HUVECs and maintained the ability to enhance macrophage ATP production, with no significant difference compared with initial DABs (Fig. S42). These structural, marker-based, and functional results indicate that DABs largely preserved their vesicular integrity, apoptotic body-related molecular composition, and key biological activities after hydrogel encapsulation and 72 h release.

To further evaluate the preliminary formulation robustness of the DABs@PHA-PVA^Gel^ platform, we assessed DABs storage stability, batch-to-batch reproducibility, and post-release bioactivity. DABs stored at − 20 °C–− 80 °C maintained a relatively stable hydrodynamic size over 30 days, supporting preliminary cryogenic storage stability (Fig. S43). Three independently prepared DABs batches showed comparable protein yield when normalized to the same parental MSCs input (Fig. S44). Moreover, DABs from different batches exhibited similar pro-proliferative effects on HUVECs, with no significant difference among batches (Fig. S45).

### Synergistic in vivo wound healing in a diabetic mouse model

**Fig. 6:**
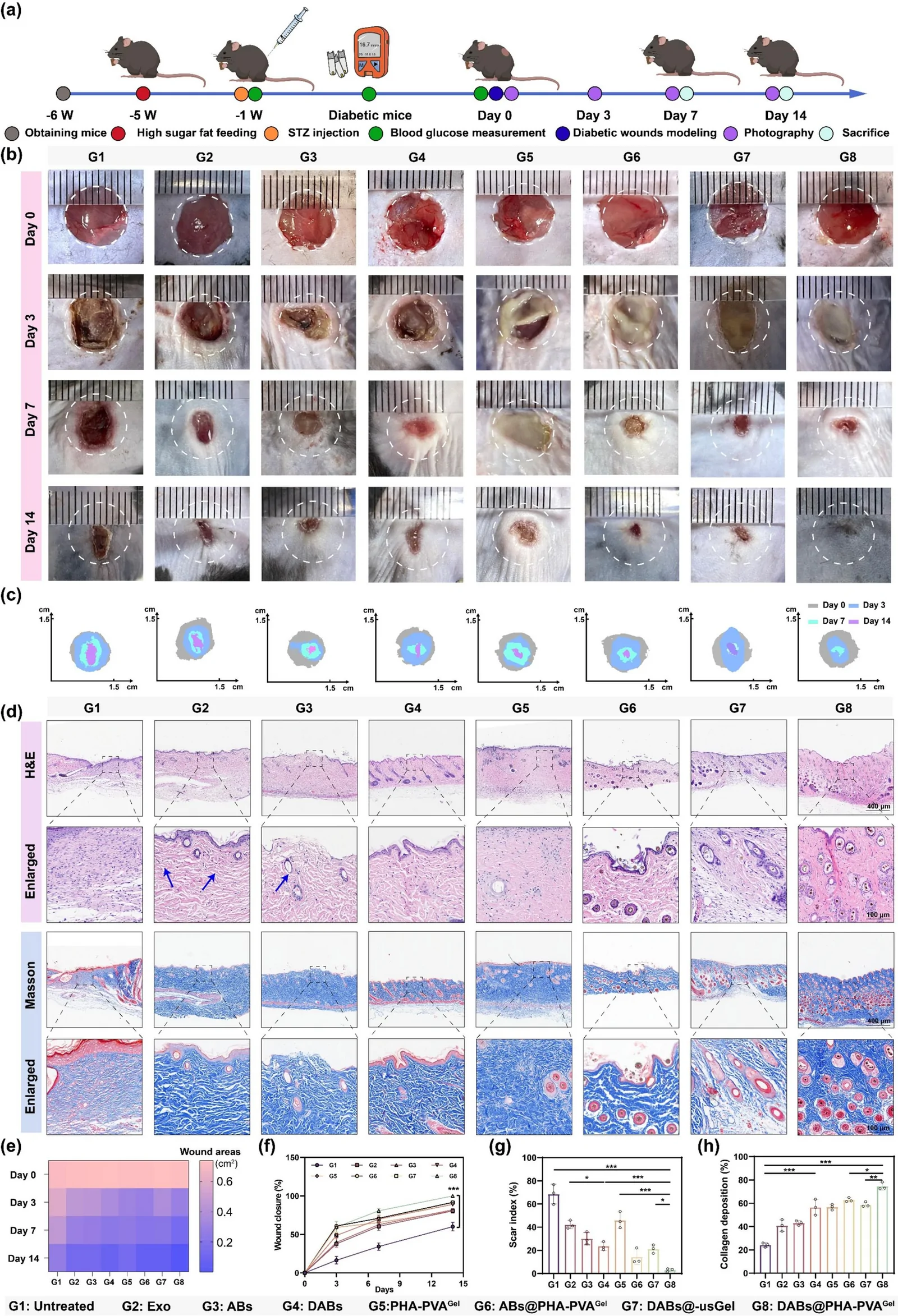
In vivo therapeutic efficacy of DABs@PHA-PVA^Gel^ in diabetic wound healing. (**a**) Schematic illustration of the treatment schedule for the diabetic full-thickness skin wound in C57 mice. (**b**) Photographs of wounds and (**c**) traces of wound closure in different treatment groups from day 0 to day 14. (**d**) Histological analysis of the wounds stained with H&E and Masson on day 14. Scale bar = 400 and 100 (enlarged) µm. (**e**) wound area (cm^2^) and (**f**) wound closure rate (*n* = 6 biologically independent wounds) over time. (**g**) Scar index and (**h**) Collagen volume fraction (*n* = 3). (*p < 0.05, **p < 0.01 ***p < 0.001.)

Following the in vitro results, we next evaluated the therapeutic performance of DABs@PHA-PVA^Gel^ in a non-splinted full-thickness diabetic excisional wound model. The experimental setup and STZ-induced diabetic model are described in Fig. 6a and Fig. S46. Mice were randomized into eight groups: untreated (G1), Exo (G2), native ABs (G3), DABs (G4), PHA-PVA^Gel^ (G5), ABs@PHA-PVA^Gel^ (G6), DABs@usGel (unresponsive hydrogel loaded with DABs, G7), and DABs@PHA-PVA^Gel^ (G8).

Wound closure was tracked over 14 days. Representative images (Fig. 6b and c) and wound area heatmaps (Fig. 6e) showed faster healing in the DABs@PHA-PVA^Gel^ group (G8), which reached a closure rate of 80.69% by day 7 (Fig. 6f). Free DABs (G4, 69.87%) produced a higher wound closure rate than free Exo (G2) under the same dosing conditions, while the further improvement observed in G8 compared with G4 likely reflects the benefit of hydrogel-mediated local retention and sustained delivery. G8 treated wounds reached complete re-epithelialization by day 14, with hair follicle regeneration already visible at the macroscopic level. This physical closure was anchored by a robust architectural recovery; H&E sections from days 7 and 14 detailed a continuous, well-consolidated epidermis packed with newly formed sweat glands and follicles (Fig. 6d, Fig. S47). This highlighted the failure of the untreated G1 group, which stalled at under 61% closure and remained structurally deficient, burdened by unresolved inflammatory infiltrates and a persistently incomplete epidermis.

Collagen accumulation reached a high level of 74.57% in the G8 tissues. This matrix accumulation showed moderate outcomes across the G7, G6, and G4 groups, with values ranging from 56.59% to 62.85%. Zooming in on the vesicle-only treatments isolates a fundamental functional gap. The DABs (G4) alone drove deposition up to 56.59% while the Exo (G2) alone stalled at 40.98% (Fig. 6h). This free-vesicle comparison suggests that DFO-programmed DABs possess stronger tissue-remodeling activity than conventional Exo under the tested conditions. Scar index results followed the same trend, with G8 achieving the lowest score (3.05%) compared to G2 (42.11%), G4 (23.62%), G5 (45.98%), and G1 (68.59%) (Fig. 6g).

### In situ mechanistic evaluation

**Fig. 7:**
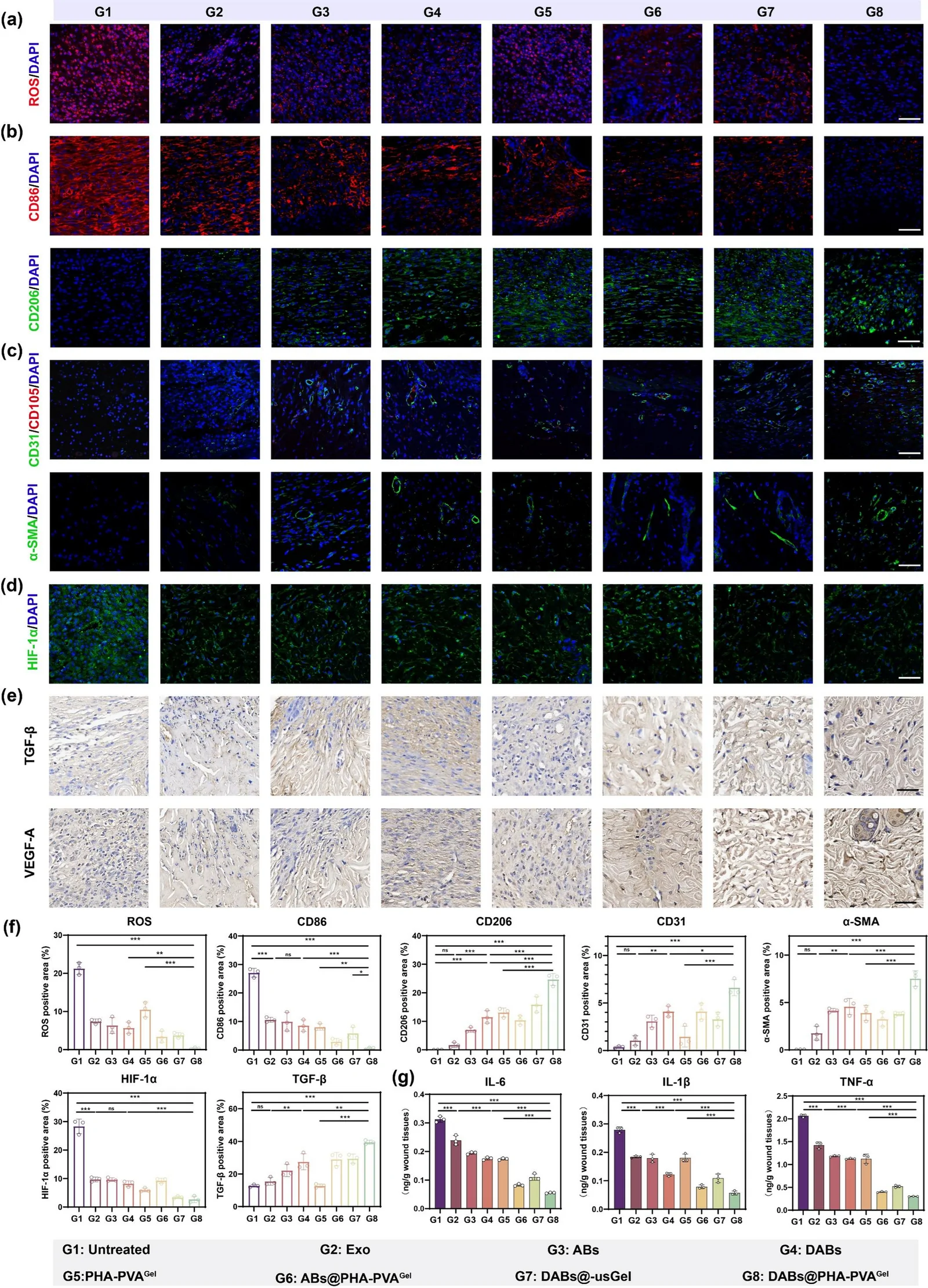
Inhibition of oxidative stress, regulation of inflammation, and promotion of vascular network reconstruction by DABs@PHA-PVA^Gel^ in diabetic wound healing. (**a**) Immunofluorescence staining for ROS (red) in tissue sections obtained from different treatment groups on day 7. (**b**) Immunofluorescence staining of the M1 macrophage marker CD86 (red) and the M2 macrophage marker CD206 (green). (**c**) Immunofluorescence staining for CD31(green)/CD105(red) and α-SMA (green) in tissue sections obtained from different treatment groups on day 7. (**d**) Immunofluorescence staining for the hypoxia-related marker HIF-1α (green) in tissue sections obtained from different treatment groups on day 7. (**e**) Immunohistochemical staining of TGF-β and VEGF-A in tissue sections obtained from different treatment groups on day 7. (Scale bar = 50 μm) (**f**) Quantification of ROS; CD86, CD206, CD31, α-SMA, HIF-1α, and TGF-β from immunofluorescence staining images and Immunohistochemical staining images (*n* = 3). (g) ELISA detection of IL-6, IL-1β, and TNF-α in wound areas after being treated with different treatment groups on day 14 (*n* = 3). *p < 0.05, **p < 0.01, ***p < 0.001

The regenerative success seen at day 14 is fundamentally rooted in earlier microenvironmental shifts. We examined wound tissues at day 7 to capture this initial remodeling phase. Excessive ROS notoriously drives persistent inflammation and impairs diabetic healing [39]. DHE staining of these early samples provided a direct readout of the underlying oxidative stress burden. Tissues from the untreated group (G1) showed intense red fluorescence, indicating high levels of oxidative stress (Fig. 7a). In stark contrast, the DABs@PHA-PVA^Gel^ group (G8) demonstrated near-complete ROS elimination (0.32% positive area), as quantified in Fig. 7f. This ROS-scavenging efficacy was significantly more potent than that of the hydrogel alone (G5: PHA-PVA^Gel^), suggesting a crucial synergistic effect between the active PHA-PVA^Gel^ carrier and the sustained, inflammation-modulating release of DABs.

This quick reduction in oxidative stress is essential for breaking the feedback cycle that sustains chronic inflammation. We therefore analyzed macrophage phenotype and distribution by immunofluorescence. As hypothesized, the G8 group showed a dramatic reduction in M1 macrophage (CD86^+^) infiltration (Fig. 7b and f). At the same time, it showed the highest levels of anti-inflammatory M2 macrophages (CD206^+^), suggesting an early phenotypic switch. This beneficial immune environment persisted until the end, as indicated by consistent immunofluorescence results at day 14 (Fig. S49 and Fig. S50). To further validate this long-term effect, we analyzed the tissue cytokine profile by ELISA at day 14. As shown in Fig. 7g and Fig. S51, the G8 group maintained the most profound reduction in pro-inflammatory cytokines (IL-6, IL-1β, TNF-α) and the highest levels of anti-inflammatory cytokines (IL-4, IL-10, VEGF-A).

Proper vascularization supports the proliferative healing phase [40]. The G8 treatment initiated this response effectively, with day 7 immunofluorescence capturing a dense CD31^+^ and α-SMA^+^ vessel network that continued expanding through day 14 (Fig. 7c and f; Fig. S52 and S53). Local protein expression aligned directly with this structural growth. Tissues receiving the G8 formulation yielded the highest concentrations of TGF-β and VEGF-A (Fig. 7e and f) and maintained these elevated pro-angiogenic signals into the later stages of repair (Fig. S54 and S55). HIF-1α expression offered a functional readout for local tissue hypoxia. Unresolved oxygen deficiency characterized the untreated G1 group since these defects maintained consistently high HIF-1α levels across the entire observation period. In contrast, the G8 group exhibited a rapid and significant decrease in HIF-1α expression (Fig. 7d and f; Fig. S56), consistent with the restoration of tissue perfusion.

Finally, the biosafety profile of DABs@PHA-PVA^Gel^ was tested to ensure clinical applicability. Histological examination of major organs and local muscle tissue, combined with serum biochemical analysis, revealed no systemic toxicity or inflammatory response (Fig. S57-S59), confirming the excellent biocompatibility of DABs@PHA-PVA^Gel^ for regenerative medicine.

### Transcriptomic analysis of DABs@PHA-PVA^Gel^-mediated diabetic wound healing

**Fig. 8:**
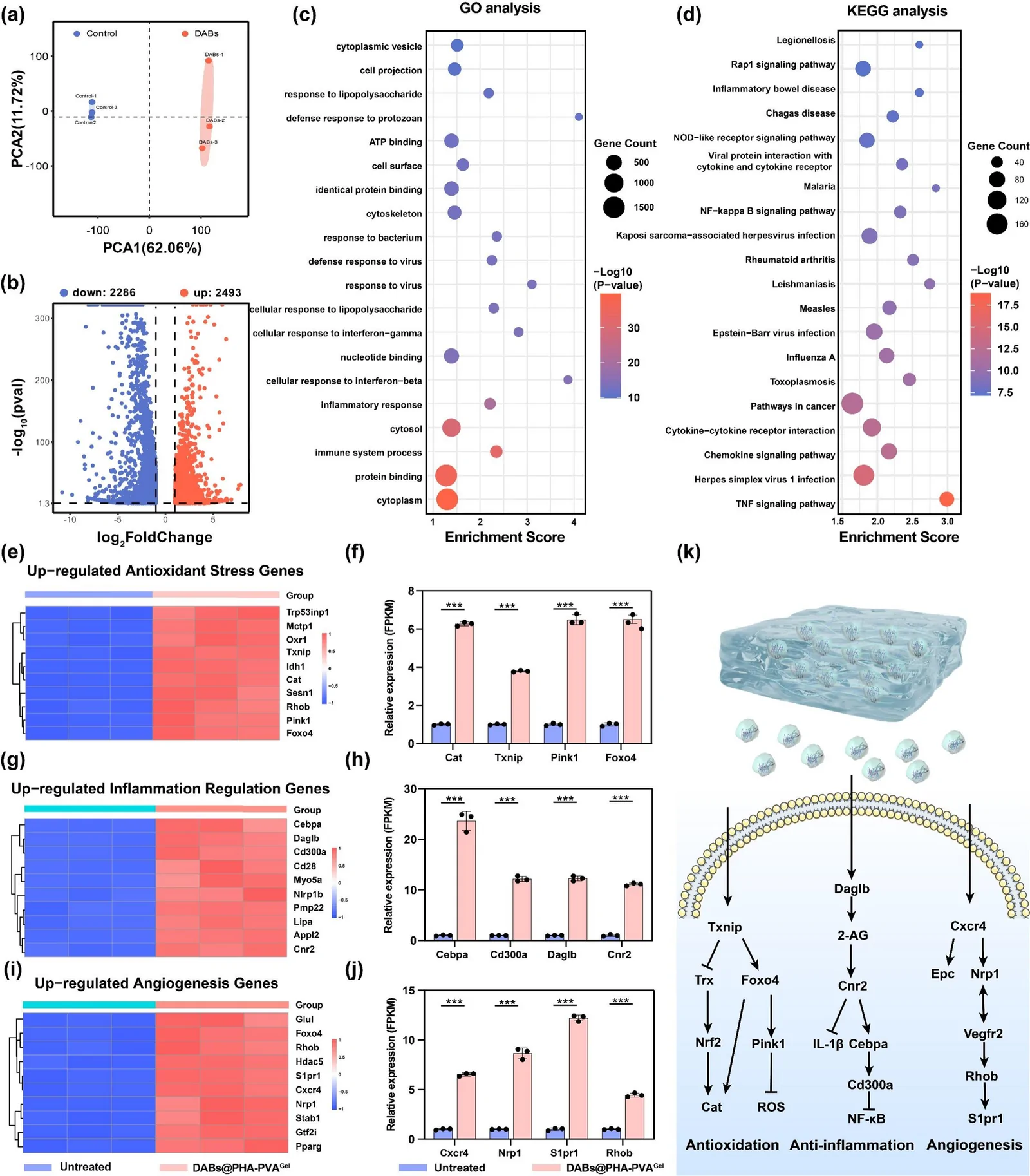
RNA-seq analysis of the diabetic wound tissue treated with DABs@PHA-PVA^Gel^. (a) Principal component analysis (PCA) of the transcriptomic profiles between the untreated and DABs@PHA-PVA^Gel^ treated groups. Each data point represents a single sample. (**b**) Volcano plots showing the upregulated (2493) and downregulated (2286) genes. (**c**) Gene ontology (GO) enrichment and (**d**) Kyoto Encyclopedia of Genes and Genomes (KEGG) pathway enrichment analysis upon 2493 differential genes and down-regulated (2286) genes. Heatmaps of significantly upregulated genes related to (**e**) antioxidant stress, (**g**) inflammation regulation, and (i) angiogenesis in the DABs@PHA-PVA^Gel^ treated groups as compared to the untreated group. Fragments per kilobase million (FPKM) of (**f**) Cat, Txnip, Pink1, and Foxo4, (**h**) Cebpa, Cd300a, Daglb, and Cnr2, (**j**) Cxcr4, Nrp1, S1pr1, and Rhob (*n* = 3). (**k**) Schematic illustration showing the potential mechanism underlying the enhancement of diabetic wound healing by the DABs@PHA-PVA^Gel^. ***p < 0.001

While histological evaluations confirmed the superior tissue quality in the DABs@PHA-PVA^Gel^ group, diabetic wound regeneration involves a complex symphony of signaling events that extends beyond structural observations. Therefore, to gain an unbiased, global understanding of the molecular reprogramming driving the regenerative effects of our system, we performed RNA-seq on wound tissues harvested on day 14 (G1: Untreated vs. G8: DABs@PHA-PVA^Gel^), with sequencing quality control and alignment statistics summarized in Supporting Tab. S1 and S2. First, Principal Component Analysis (PCA) demonstrated that the untreated (G1) and DABs@PHA-PVA^Gel^-treated (G8) groups segregated into distinct, non-overlapping clusters (Fig. 8a). Differential expression analysis identified 4779 differentially expressed genes (DEGs) between the two groups (FDR < 0.05, |log2FC| > 1), comprising 2493 upregulated and 2286 downregulated genes in the G8 group, as visualized in the volcano plot (Fig. 8b).

GO and KEGG enrichment analyses were performed to evaluate the biological pathways associated with these DEGs. GO analysis indicated that the downregulated genes in the G8 group were primarily enriched in pro-inflammatory biological processes, including ‘response to interferon-gamma’, ‘inflammatory response’, and ‘cytokine response’ (Fig. 8c and Fig. S60). Conversely, the upregulated genes were primarily associated with regenerative and metabolic processes, such as ‘cell projection’, ‘cytoskeleton organization’, and ‘ATP binding’. KEGG pathway analysis confirmed a suppression of classical inflammatory networks in the treated tissues. Specific cascades like TNF, NF-κB, and general cytokine signaling were distinctly underrepresented. This dampened inflammatory state allowed tissue repair and metabolic programs to dominate the enrichment profiles, with Foxo and Rap1 signaling emerging as the primary driven pathways (Fig. 8d and Fig. S61).

To further examine transcriptional changes corresponding to the wound healing, we analyzed targeted gene sets associated with oxidative stress, inflammation, and angiogenesis [41–43]. Heatmaps demonstrated a coordinated upregulation of antioxidant genes (e.g., Trp53inp1, Foxo4, Pink1), immunomodulatory genes (e.g., Cebpa, Cd300a, Cnr2), and pro-angiogenic genes (e.g., Cxcr4, Nrp1, S1pr1) in the G8 group relative to G1 (Fig. 8e and g, and 8i). The expression levels (FPKM) of representative hub genes were plotted to quantify these cluster trends (Fig. 8f and h, and 8j). Compared to the untreated control, the DABs@PHA-PVA^Gel^ treatment resulted in significantly higher expression levels of specific antioxidant (Cat, Txnip, Foxo4), anti-inflammatory (Cebpa, Cd300a, Cnr2), and pro-angiogenic (Cxcr4, Nrp1, Rhob) genes. Together, these targeted transcriptional analyses provide experimental validation of the RNA-seq-identified repair-related pathways, demonstrating that DABs@PHA-PVA^Gel^ coordinately regulates antioxidant defense, inflammatory resolution, and angiogenic responses at the gene expression level.

Based on these transcriptomic findings, we tentatively interpret a molecular mechanism (Fig. 8k). These mechanisms suggest that DABs@PHA-PVA^Gel^ may influence multiple biological processes rather than a single dominant pathway. Alterations in Txnip, Foxo4, and Pink1 expression are consistent with modulation of Pink1-mediated mitochondrial quality control, which may contribute to improved redox balance [44, 45]. In parallel, increased expression of genes within the Daglb/Cnr2 axis may reflect engagement of endogenous pathways linked to inflammation resolution, which have recently been implicated in skin repair processes [46, 47]. The activation of the Cxcr4/Nrp1/S1pr1 axis points to a coordinated vascular repair mechanism, where Cxcr4 recruits endothelial progenitor cells and Nrp1 and S1pr1 support Vegfr2 signaling and Rhob GTPase activation to drive endothelial migration and vessel maturation [48, 49].

To further validate the RNA-seq-derived pathway interpretation, we performed qPCR and Western blot analysis of representative genes and proteins involved in the Txnip/Foxo4, Daglb/Cnr2, and Cxcr4/Nrp1/S1pr1 axes. qPCR analysis showed that Txnip, Foxo4, Daglb, Cnr2, Cxcr4, Nrp1, and S1pr1 were significantly upregulated in the DABs@PHA-PVA^Gel^ group compared with the untreated group. Consistently, Western blot analysis confirmed increased protein expression of Txnip, Foxo4, Daglb, Cnr2, Cxcr4, Nrp1, and S1pr1 in DABs@PHA-PVA^Gel^-treated wound tissues. These results support the key RNA-seq-identified pathways and further indicate the involvement of antioxidant regulation, anti-inflammatory endocannabinoid signaling, and angiogenic remodeling in DABs@PHA-PVA^Gel^-mediated diabetic wound repair (Fig. S62 and Fig. S63).

## Discussion

Clinical translation of MSC-derived exosomes remains limited by their relatively low natural secretion yield and the instability of their therapeutic performance under pathological conditions [1, 4, 5, 50]. Apoptotic bodies (ABs) offer a high-yield alternative and possess intrinsic immunomodulatory properties largely attributed to phosphatidylserine (PS) exposure on their surface [6, 7, 51]. However, their native pro-angiogenic activity is generally insufficient to meet the substantial vascularization demands of severely ischemic environments such as diabetic wounds [10, 11, 52, 53]. In the present study, we demonstrate that pre-programming MSCs with DFO prior to apoptosis enables the generation of programmed vesicles (DABs) that effectively address both limitations.

DABs displayed enhanced biological activity that can be mechanistically linked to DFO-induced remodeling of their regulatory miRNA cargo [54, 55], our work reveals an additional effect that applying DFO prior to apoptosis induction fundamentally alters the vesicle’s cargo composition. For instance, DABs were found to be heavily loaded with specific miRNAs known to influence angiogenesis and cell migration such as hsa-miR-4674, hsa-miR-3976, and hsa-miR-320e [56]. The levels of these molecules far exceeded what we observed in typical MSC-derived exosomes or native apoptotic bodies. These altered miRNAs likely contribute to the superior pro-angiogenic and immunomodulatory effects observed in our study. However, DABs should not be viewed as single-component miRNA carriers. Although this study focused on miRNA cargo because small RNA-seq revealed pronounced DFO-induced remodeling of pro-regenerative miRNAs, apoptotic vesicles also contain mRNAs, proteins, lipids, and cellular/organelle-associated components.

Surface exposure of PS is known to facilitate macrophage efferocytosis [57, 58], a property that is retained in DABs. Analysis of recipient macrophages suggests the involvement of additional regulatory mechanisms beyond canonical pathways. The miRNA repertoire delivered by DABs appears to influence mitochondrial energy metabolism, as evidenced by increased cellular ATP production. To date, most EV-based therapeutic strategies have focused on modulating inflammatory signaling [59, 60], whereas modulation of mitochondrial metabolism in recipient immune cells by programmed apoptotic bodies has not been reported. Whether this metabolic shift directly drives the M1-to-M2 polarization observed in our study remains unclear. However, given the well-established dependence of M2 macrophages on oxidative phosphorylation [61], this association merits further investigation.

To improve vesicle stability within the hostile microenvironment of diabetic wounds, DABs were further encapsulated in a glucose- and ROS-responsive PHA-PVA^Gel^. The in vivo results showed that DABs@PHA-PVA^Gel^ effectively promoted early wound closure among the tested formulations, including free Exo, free DABs, PHA-PVA^Gel^, ABs@PHA-PVA^Gel^, and DABs@usGel. We then performed RNA-seq analysis on the treated wound tissues to get a clearer picture of the biological changes driving this accelerated healing. The transcriptomic profiles showed a few distinct but coordinated trends. We noted a drop in the Txnip/Foxo4 cascade alongside the restoration of Nrf2-driven redox homeostasis [44, 45]. At the same time, the Daglb/Cnr2 signaling axis was upregulated that makes sense given the emerging role of cannabinoid receptor 2 in clearing up skin inflammation [46, 47]. Furthermore, there was an upregulation across the Cxcr4/Nrp1/S1pr1 receptor network, which is known to recruit endothelial progenitor cells for vascular repair [48, 49]. The simultaneous modulation of these pathways by a single vesicle-based intervention is noteworthy and likely reflects the diverse regulatory capacity of the miRNAs carried by DABs.

Despite these encouraging findings, our current study does have a few constraints. Although the STZ-induced diabetic mouse model reproduces certain aspects of diabetic pathology, it does not fully capture the complex systemic complications present in human type 2 diabetes [62, 63]. In addition, murine wound healing relies heavily on contraction, whereas re-epithelialization plays a more dominant role in human chronic wounds [64, 65]. In the present study, a non-splinted full-thickness diabetic wound model was selected to preserve direct hydrogel–wound bed interaction and avoid potential interference from splint-associated suturing, mechanical constraint, foreign-body contact, and local inflammation during repeated hydrogel dressing replacement. Because all treatment groups were evaluated under identical wound conditions, the relative comparison among the tested formulations remains internally consistent. From a formulation perspective, DABs@PHA-PVA^Gel^ was designed as an on-demand injectable and in situ-forming hydrogel dressing rather than a pre-formed DABs-loaded hydrogel intended for frozen storage and thawing before use. This design enables DABs to be mixed with the hydrogel precursor solutions immediately before application, allowing the formulation to conformally contact the irregular wound bed after in situ gelation. The 72 h DABs release profile was matched with the 3-day dressing replacement schedule used in vivo, which is consistent with periodic topical wound-dressing management. During each replacement, the residual hydrogel was gently removed under aseptic conditions before fresh formulation application. In addition, we provided preliminary evidence for 30-day cryogenic size stability of DABs, batch-to-batch consistency of DABs yield and HUVEC pro-proliferative activity, and preservation of DABs integrity and functionality after 72 h release from PHA-PVA^Gel^. These results support the formulation feasibility and practical applicability of DABs@PHA-PVA^Gel^ as an on-demand injectable hydrogel dressing for diabetic wound repair.

In conclusion, our findings demonstrate the potential of upstream cellular programming as a practical strategy to generate functionally enhanced apoptotic vesicles. Notably, a single preconditioning step using an approved small molecule was sufficient to substantially reshape apoptotic vesicle cargo, conferring both immunoregulatory and pro-angiogenic properties. Moreover, the fact that these programmed vesicles might actively alter recipient cell metabolism opens a new therapeutic angle worth exploring. This strategy could extend well beyond diabetic wound repair. It may also hold promise for other conditions in which scalable, programmable vesicle-based therapies are needed, such as peripheral nerve injuries, inflammatory skin disorders, and ischemic vascular diseases.

## Supplementary Information

Below is the link to the electronic supplementary material.

Supplementary Material 1

Supplementary Material 2

## Data Availability

No datasets were generated or analysed during the current study.
