# Supplementary material for: Responsive hydrogel delivery of DFO-programmed apoptotic bodies drives redox-immuno-angiogenic remodeling in diabetic wounds
Source: J Nanobiotechnology. 2026 Jul 22;24:964. doi: 10.1186/s12951-026-04817-0 (PMC13673864; doi:10.1186/s12951-026-04817-0)
Supplement: Supplementary file 1 [file 12951_2026_4817_MOESM1_ESM.docx]

Supporting Information

**Responsive Hydrogel Delivery of DFO-Programmed Apoptotic Bodies Drives Redox-Immuno-Angiogenic Remodeling in Diabetic Wounds**

Hao Guo ^a,1^, Jian Li ^a,1^, Chengcheng Gu ^a^, Ning Liu ^a^, Jie Sun ^a^, Danhui Wang ^a^, Siyuan Meng ^a^, Dongshu Geng ^a^, Yunfei Lian^a,^ ^b, *^, Fangnan Lv ^a, *^, Meirong Huo ^a, *^

^a^ Department of Pharmaceutics, China Pharmaceutical University, Nanjing 211198, China.

^b^ Nanjing Sairicon Biomedical Technology Co., Ltd., Nanjing 210039, China

* Corresponding authors.

E-mail address: lyf_snpharma@126.com (Y. Lian), lvfangnan@126.com (F. Lv), huomeirongcpu@163.com (M. Huo)

^1^These authors contributed equally to this work.

Supporting Figures

**Fig. S1.** Flow cytometric characterization of MSC surface markers.

**Fig. S2.** Effect of DFO concentration on MSCs cell viability (n = 3).

**Fig. S3.** Optimization of DFO concentration for HIF-1α stabilization in MSCs before apoptosis induction. (a) Western blot analysis of HIF-1α expression in MSCs treated with different concentrations of DFO. β-Actin was used as the loading control. (b) Quantitative analysis of HIF-1α expression in DFO-treated MSCs. Data are presented as mean ± SD. ns, not significant; ****p* < 0.001.

**Fig. S4.** Morphological changes of MSCs induced by UV irradiation at different time (Scale bar = 100 μm).

**Fig. S5.** Cell apoptosis analysis of MSCs after 10 min UV-induced culture for 24h. (n = 3). (a) Cell apoptosis of MSCs after 10 min UV-induced culture for 24 h by flow cytometry. (b) Quantitative analysis of MSCs apoptosis cells after 10 min UV-induced culture for 24 h (n = 3). ****p* < 0.001.

**Fig. S6.** HIF-1α expression in MSCs after DFO pretreatment followed by UV-induced apoptosis. MSCs were pretreated with different concentrations of DFO for 24 h, washed with PBS, subjected to UV irradiation, and cultured for another 24 h under apoptosis-induction conditions before analysis. (a) Western blot analysis of HIF-1α expression. GAPDH was used as the loading control. (b) Quantitative analysis of HIF-1α expression after apoptosis induction. Data are presented as mean ± SD. ns, not significant; ****p* < 0.001.

**Fig. S7.** Schematic of the procedure used for the isolation of ABs and DABs via sequential centrifugation.

**Fig. S8**. Comparative analysis of phosphatidylserine exposure in Exo, ABs, and DABs. Exo, ABs, and DABs were stained with Annexin V-FITC and analyzed by flow cytometry.

**Fig. S9.** Western blot analysis of apoptotic body-related markers in ABs and DABs.

**Fig. S10.** Comparative Western blot analysis of exosome-associated markers in Exo, ABs, and DABs. Equal amounts of total vesicular protein from Exo, ABs, and DABs were loaded for each lane. Hsp70, CD63, TSG101, CD9, and Calnexin were detected using an exosome identification kit.

**Fig. S11**. Autofluorescence controls for vesicle uptake analysis in HUVECs and RAW264.7 cells. Representative confocal laser scanning microscopy images of untreated HUVECs (a) and RAW264.7 cells (b) acquired under the same imaging settings used for DiI-labeled vesicle uptake analysis. Scale bar = 10 μm.

**Fig. S12.** Cellular uptake of ABs and DABs by HaCaT cells. (a) Representative confocal laser scanning microscopy images of HaCaT cells after incubation with DiI-labeled ABs or DABs for 4 h. Untreated cells were included as the autofluorescence control. Scale bar = 10 μm. (b) Flow cytometric analysis of HaCaT cells after incubation with DiO-labeled ABs or DABs for 4 h.

**Fig. S13.** Cellular uptake of HaCaT, HUVEC, and RAW264.7 cells after being treated with ABs and DABs for 4 h by flow cytometry (n = 3). ****p* < 0.001.

**Fig. S14.** Expression of the differential miRNAs that are significant to diabetic wound regeneration, including (a) positive regulation of angiogenesis and (b) positive regulation of cell migration (n = 5). ***p* < 0.01, ****p* < 0.001.

**Fig. S15**. GO and KEGG enrichment analyses of predicted target genes for the top 10 upregulated miRNAs in DABs. (a) GO biological process enrichment analysis of predicted target genes. (b) KEGG pathway enrichment analysis of predicted target genes. Enriched terms were ranked by −log10(P value).

**Fig. S16.** PPI network showing the interaction relationship among the predicted target genes (colored by miRNA category) of the 10 significantly upregulated miRNAs in DABs, corresponding to the hub gene networks identified by the MCODE algorithm.

**Fig. S17.** Particle size distribution of exosomes.

**Fig. S18.** Identification of exosomes. (a) TEM morphological characterization of exosomes (Scale bar = 50 nm). (b) WB identification of exosome surface markers CD9, CD63, TSG101, and Hsp70.

**Fig. S19.** Volcano plot of differentially expressed miRNAs (Exo vs ABs).

**Fig. S20.** Clustering heatmap of differentially expressed miRNAs (Exo vs ABs).

**Fig. S21.** Volcano plot of differentially expressed miRNAs (Exo vs DABs).

**Fig. S22.** Clustering heatmap of differentially expressed miRNAs (Exo vs DABs).

**Fig. S23.** Venn diagram of differentially expressed miRNAs in Exo, ABs, and DABs.

**Fig. S24.** Chord diagram showing the functional associations between the predicted target genes of the top 10 upregulated miRNAs in DABs relative to Exosomes and inflammation-related biological processes, including inflammatory response, cytokine production, leukocyte migration, NF-κB signaling, macrophage activation, and acute inflammatory response.

**Fig. S25.** Chord diagram showing the functional associations between the predicted target genes of the top 10 upregulated miRNAs in DABs relative to Exosomes and tissue repair functions, including cell migration, cell adhesion, angiogenesis, epithelial proliferation, extracellular matrix (ECM) organization, and wound healing.

**Fig. S26.** DABs facilitated the migration of HaCaT. (a) Representative images of scratch assays in HaCaT treated with Exo, ABs, and DABs. (b) Cell migration of HaCaT cells treated with different formulations for 24 h. (n = 3) **p* < 0.05, ***p* < 0.01, ****p* < 0.001.

**Fig. S27.** DABs facilitated the proliferation of HUVECs (n = 3). ***p* < 0.01, ****p* < 0.001.

**Fig. S28**. JC-1 staining of RAW264.7 after different treatments. (a) Representative JC-1 staining images. Scale bar = 20 μm. (b) Quantification of the JC-1 red/green fluorescence intensity ratio. Data are presented as mean ± SD. ns, not significant; **p* < 0.05, ****p* < 0.001.

**Fig. S29**. OCR/ECAR analysis of RAW264.7 after different treatments. (a) Basal oxygen consumption rate (OCR). (b) FCCP-stimulated OCR. (c) Baseline extracellular acidification rate (ECAR). Data are presented as mean ± SD. **p* < 0.05, ***p* < 0.01, ****p* < 0.001.

**Fig. S30**. Intracellular ROS detection in RAW264.7 macrophages after different treatments. (a) Representative DCFH-DA staining images. Scale bar = 20 μm. (b) Quantification of ROS fluorescence intensity. Data are presented as mean ± SD. ns, not significant; **p* < 0.05, ***p* < 0.01, ****p* < 0.001.

**Fig. S31.** UV-vis spectra of HA and PHA. PHA exhibited a characteristic absorption peak at ~280 nm, indicative of the phenyl ring from PBA.

**Fig. S32.** ^1^H-NMR (D_2_O) spectra of PHA. Distinct aromatic proton signals appearing at 7.0–8.0 ppm were consistent with aminophenylboronic acid, confirming successful PBA incorporation.

**Fig. S33.** FT-IR spectra of HA and PHA. Characteristic absorption bands at 1647 cm^-1^ (amide) and 1350 cm^-1^ (O–B–O) appeared in PHA but were absent in native HA.

**Fig. S34.** Photographs showed the gelation of the hydrogels.

**Fig. S35.** The morphology of hydrogel characterized by SEM. (Scale bar = 100 μm)

**Fig. S36.** Representative Live/Dead staining images of HUVEC (Green: live; Red: dead) following exposure to H_2_O_2_. The results demonstrate that PHA-PVA^Gel^ effectively prevents H_2_O_2_-induced cell death, exhibiting superior cytoprotective capability compared to the HA-PVA^Gel^ and H_2_O_2_-only groups (Scale bars = 200 μm).

**Fig. S37.** The effect of PHA-PVA^Gel^ on the cell viability of HaCaT and HUVEC at (a) 24 h, (b) 48 h, and (c) 72 h (n = 3). ***p* < 0.01, ****p* < 0.001.

**Fig. S38**. Higher-magnification orthogonal confocal image showing the Z-axis distribution of DiI-labeled DABs within PHA-PVA^Gel^.

**Fig. S39**. TEM characterization of DABs after 72 h release from PHA-PVA^Gel^. Scale bar = 100 nm.

**Fig. S40**. Annexin V-FITC analysis of PS exposure in DABs after 72 h release from PHA-PVA^Gel^.

**Fig. S41**. Western blot analysis of apoptotic body-related markers in DABs after 72 h release from PHA-PVA^Gel^.

**Fig. S42**. Functional activity of DABs after 72 h release from PHA-PVA^Gel^. (a) HUVEC proliferation after treatment with initial DABs or released DABs. (b) Macrophage ATP production after treatment with initial DABs or released DABs. Data are presented as mean ± SD. ns, not significant; ***p* < 0.01; ****p* < 0.001.

**Fig. S43**. Preliminary storage stability of DABs. Data are presented as mean ± SD.

**Fig. S44**. Batch-to-batch reproducibility of DABs yield. Data are presented as mean ± SD. ns, not significant.

**Fig. S45**. Batch-to-batch consistency of DABs bioactivity. Data are presented as mean ± SD. ns, not significant; ****p* < 0.001.

**Fig. S46.** Changes in blood glucose levels in mice.

**Fig. S47.** Representative H&E staining images of the wound area at day 7 after the first treatment (Scale bar = 400 μm and 100 μm).

**Fig. S48.** Representative Masson staining images of the wound area at day 7 after the first treatment (Scale bar = 400 μm and 100 μm).

**Fig. S49.** Immunofluorescence staining of CD86 in wound areas on day 14 (Scale bar = 50 μm).

**Fig. S50.** Immunofluorescence staining of CD206 in wound areas on day 14 (Scale bar = 50 μm).

**Fig. S51.** ELISA detection of IL-4, IL-10, and VEGF-A in wound areas after being treated with various formulations (n=3). **p* < 0.05, ***p* < 0.01, ****p* < 0.001

**Fig. S52.** Immunofluorescence staining of CD31 (Green)/CD105 (Red) in wound areas after being treated with various formulations on day 14 (Scale bar = 50 μm).

**Fig. S53.** Immunofluorescence staining of α-SMA in wound areas after being treated with various formulations on day 14 (Scale bar = 50 μm).

**Fig. S54.** Immunohistochemistry staining of TGF-β in wound areas after being treated with various formulations on day 14 (Scale bar = 50 μm).

**Fig. S55.** Immunohistochemistry staining of VEGF-A in wound areas after being treated with various formulations on day 14 (Scale bar = 50 μm).

**Fig. S56.** Immunofluorescence staining of HIF-1α in wound areas after being treated with various formulations on day 14 (Scale bar = 50 μm).

**Fig. S57.** H&E images of major organs (heart, liver, spleen, lung, and kidney) after being treated with untreated group and DABs@PHA-PVA^Gel^ group (Scale bar = 200 μm).

**Fig. S58.** Liver (A, B, C, and D) and kidney (E and F) functions of serum of mice in the saline group and DABs@PHA-PVA^Gel^ group (n=3).

**Fig. S59.** H&E staining images of muscle after intramuscular injection of ABs and DABs for 7 days (Scale bar = 200 μm).

**Fig. S60.** GO enrichment map of differentially expressed genes between the untreated group and DABs@PHA-PVA^Gel^.

**Fig. S61.** KEGG enrichment analysis of differentially expressed genes between the untreated group and DABs@PHA-PVA^Gel^.

**Fig. S62**. qPCR validation of RNA-seq-identified key genes in wound tissues. Data are presented as mean ± SD. ***p* < 0.01, ****p* < 0.001.

**Fig. S63**. Western blot validation of RNA-seq-identified key pathway proteins in wound tissues. (a) Western blot analysis of Txnip and Foxo4. (b) Western blot analysis of Daglb and Cnr2. (c) Western blot analysis of Cxcr4, Nrp1, and S1pr1. (d–f) Quantitative analysis of relative protein expression. Data are presented as mean ± SD. ****p* < 0.001.

Supporting Tables

**Tab. S1** Sequencing data statistic.

| Sample | Clean reads | | Clean bases | GC content | ≥Q 30 |
| --- | --- | --- | --- | --- | --- |
| Control-1 | 88931236 | 13288875885 | | 50.13% | 93.69% |
| Control-2 | 90291982 | 13492981620 | | 50.10% | 93.56% |
| Control-3 | 48247830 | 7208747182 | | 49.94% | 93.63% |
| DABs@PHA-PVA^Gel^-1 | 81645292 | 12208734520 | | 50.35% | 93.49% |
| DABs@PHA-PVA^Gel^-2 | 95290442 | 142394823 48 | | 50.34% | 93.42% |
| DABs@PHA-PVA^Gel^-3 | 75713030 | 11316612860 | | 50.27% | 93.51% |

**Tab. S2** Statistics of sequence alignment results of the sample sequencing data and the selected reference genome.

| Sample | Total Reads | Mapped reads rates | Unique mapped reads rates |
| --- | --- | --- | --- |
| Control-1 | 88764288 | 98.80% | 94.10% |
| Control-2 | 90143556 | 98.60% | 93.90% |
| Control-3 | 48164212 | 98.50% | 93.70% |
| DABs@PHA-PVA^Gel^-1 | 81452778 | 98.80% | 94.40% |
| DABs@PHA-PVA^Gel^-2 | 95065854 | 98.70% | 94.30% |
| DABs@PHA-PVA^Gel^-3 | 75499548 | 98.80% | 94.60% |

**Tab. S3**. Primer sequences used for RT-qPCR analysis of wound tissues.

| Gene | Forward sequence (5’-3’) | Reverse sequence (5’-3’) |
| --- | --- | --- |
| Txnip | ATACCCCTGACCTAATGGCAC | TTCGAGCAGAGACTGACACAC |
| Foxo4 | TCATCAGCCAGGCCATTGAA | TTGTGACGGATGGAGTTCTTC |
| Daglb | TGTGTCAGCATGAGAGGAACC | CCAGAGAAGCCCAGACCATC |
| Cnr2 | ACTCATCTGCGAAAGTGTGAGA | GTCCCAGAAGACTGGGTGTCATT |
| Cxcr4 | GCCATGGAACCGATCAGTGTG | TTGCCGACTATGCCAGTCAA |
| Nrp1 | GCTGTGAAGTGGAAGCACC | CTGGAAGTCATCACCTGTGC |
| S1pr1 | TCAGGGAACTTTGCGAGTGA | GAAACAGCAGCCTCGCTCA |
| Gapdh | AGGTCGGTGTGAACGGATTTG | GAAGGGGTCGTTGATGGCAA |
